# Response functions for electrically coupled neuronal network: a method of local point matching and its applications

**DOI:** 10.1007/s00422-016-0681-y

**Published:** 2016-03-18

**Authors:** Lu Yihe, Yulia Timofeeva

**Affiliations:** Centre for Complexity Science, University of Warwick, Coventry, CV4 7AL UK; Department of Computer Science and Centre for Complexity Science, University of Warwick, Coventry, CV4 7AL UK

**Keywords:** Dendrites, Gap junctions, Network dynamics, Sum-over-trips

## Abstract

Neuronal networks connected by electrical synapses, also referred to as gap junctions, are present throughout the entire central nervous system. Many instances of gap-junctional coupling are formed between dendritic arbours of individual cells, and these dendro-dendritic gap junctions are known to play an important role in mediating various brain rhythms in both normal and pathological states. The dynamics of such neuronal networks modelled by passive or quasi-active (resonant) membranes can be described by the Green’s function which provides the fundamental input-output relationships of the entire network. One of the methods for calculating this response function is the so-called ‘sum-over-trips’ framework which enables the construction of the Green’s function for an arbitrary network as a convergent infinite series solution. Here we propose an alternative and computationally efficient approach for constructing the Green’s functions on dendro-dendritic gap junction-coupled neuronal networks which avoids any infinite terms in the solutions. Instead, the Green’s function is constructed from the solution of a system of linear algebraic equations. We apply this new method to a number of systems including a simple single cell model and two-cell neuronal networks. We also demonstrate that the application of this novel approach allows one to reduce a model with complex dendritic formations to an equivalent model with a much simpler morphological structure.

## Introduction

Neuronal cells have a distinctive structure which differentiates them from any other cell types. The most extended parts of many neurons are dendrites, and their morphological complexity has fascinated scientists since the exemplary work of Ramón y Cajal [3]. Organised in a network, neurons receive and integrate thousands of neuronal inputs via both chemical and electrical synapses located primarily on dendrites. With the development of sharp micropipette electrodes, dynamic properties of dendritic membranes started to be revealed through intracellular recordings, and in the late 1950s experimental work was complemented with the pioneering theoretical work of Wilfrid Rall on the application of cable theory to dendritic modelling. Rall’s significant contribution to the topic of dendritic function is nicely summarised in the book of Segev et al. [15]. Recent experimental and theoretical/computational studies at a single cell level reinforce the fact that dendritic morphology combined with membrane properties plays an important role in dendritic integration (two books, edited by Stuart et al. [16] and Cuntz et al. [7], give informative overviews from both an experimental and a theoretical perspective). An additional level of complexity associated with synaptic connectivity needs to be taken into consideration when dynamics of neuronal networks, rather than single cell dynamics, are investigated.

The dendritic membrane of various types of neurons is known to be equipped with voltage-gated ion channels, nonuniformly distributed throughout dendritic arbours and often demonstrating nonlinear dynamics. Many models of neuronal cells with retention of complex dendritic formations are built by combining the linear (passive) properties of dendrites together with nonlinear (active) dynamics of ion channels. At the level of a single cell or at the network level, such models are restricted to being solved only by numerical methods, based on a compartmental approach [14]. Although the nonlinear properties of voltage-gated ion channels contribute considerably to neuronal input-output relations, it is important to recognise that the purely passive or resonant (quasi-active) properties of dendritic membranes provide the fundamental core for signal filtration and integration. Resonant dynamics of dendritic membrane are usually associated with the hyperpolarisation-activated $I_h$ current and, from a mathematical perspective, can be described by linearising channel kinetics [9–11].

Here we focus on a network of neuronal cells with purely passive or resonant membrane dynamics coupled by dendro-dendritic electrical synapses, also known as gap junctions. Gap junctions are mechanical and electrically conductive links between adjacent neuronal cells that permit direct electrical connections between them. Having been first discovered at the giant motor synapses of the crayfish in the late 1950s, gap junctions are now known to be expressed in the majority of cell types in the brain [8, 13]. Using the cable theory approach for modelling dendritic arbours, the response of an entire dendro-dendritic gap junction-coupled neuronal network to any injected current can be represented by a response function. This response function, often referred as a Green’s function, describes the voltage dynamics along a network structure in response to a Dirac delta pulse applied at a given discrete location. One of the methods for constructing the Green’s function, the so-called ‘sum-over-trips’ approach, is built on a path integral formulation and was originally proposed by Abbott et al. [1, 2] for passive dendrites of a single cell and then generalised by Coombes et al. [6] for resonant membranes and Timofeeva et al. [19] for a neuronal network. This method calculates the response function as a convergent infinite series solution consisting of terms with various trips (paths) on a given branching structure and the associated coefficients obtained by the sum-over-trips rules. It has been shown at the single cell level that although convergence of this method is fast for simplified dendritic structures, the number of trips to guarantee a small convergence error for real morphologies might be large and have a strong effect on computational efficiency [4]. Here we propose an alternative method for calculating the Green’s function on a neuronal network coupled by dendro-dendritic gap junctions. This new method, named as a method of local point matching, is inspired by the sum-over-trips approach and utilises the trip coefficients of that method, but avoids the construction of any trips. Instead, the new method is based on the construction of a linear system of algebraic equations and therefore leads to compact solutions without an infinite number of terms.

In Sect. 2, we introduce the network model for gap junction-coupled neurons. Each neuron in the network consists of a soma and a dendritic arbour. Cellular membrane dynamics are modelled by a resonant electrical circuit. In Sect. 3, we develop a new method of local point matching from the generalised form of the sum-over-trips approach [19] for constructing the Green’s function for an arbitrary network. Applications of this new method are demonstrated in Sect. 4. We start with a simple single cell model consisting of a soma and dendrite and then move to a two-cell simplified network and, finally, to a more complex tufted network. Not only do we apply the local point matching method for constructing the Green’s functions in each case, but also use it to reduce the full two-cell tufted network model to an equivalent and much simpler model. The last two aforementioned sections include the key results and skip some mathematical details on the derivation of analytical results. We refer the interested reader to “Appendix” for detailed mathematical derivations. Finally, in Sect. 5, we provide a discussion of our results, as well as possible extensions of this work.

## The model

**Fig. 1:**
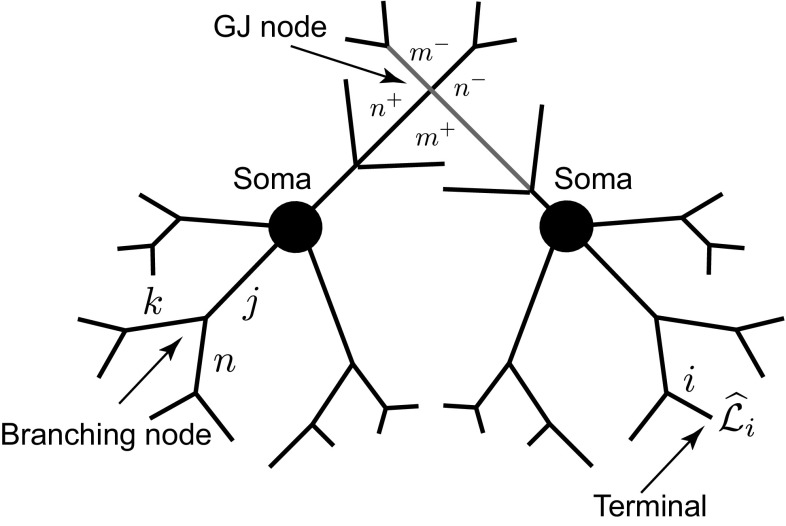
A network of two cells connected by a gap-junctional (GJ) node

We consider a network of neuronal cells. Each cell consists of an arbitrary structure of a dendritic morphology and a lumped soma, and cells in the network are connected by gap junctions (see an illustrative example for two cells in Fig. 1).

The transmembrane voltage $V_i(X, t)$ on an individual branch *i* of each cell is governed by the following set of equations:

1 $$\begin{aligned}&\frac{\partial V_i}{\partial t}=D_i\frac{\partial ^2 V_i}{\partial X^2}-\frac{V_i}{\tau _i}-\frac{1}{C_i}\left[ I_i-I_{\mathrm{inj},i} \right] , \end{aligned}$$

2 $$\begin{aligned}&L_i\frac{\partial I_i}{\partial t}=-r_iI_i+V_i, \qquad 0 \le X \le {\widehat{\mathcal {L}}}_i, \qquad t\ge 0. \end{aligned}$$

These equations provide a more general case of the linear cable theory with the cell membrane modelled by the so-called ‘LRC’ (or resonant) circuit instead of the ‘RC’ (or purely passive) circuit. The resonant circuit for each branch is described by the specific membrane capacitance $C_i$, the resistance across a unit area of passive membrane $R_i$ and an inductance $L_i$ in series with a resistance $r_i$. The presence of an inductive path in the circuit is the result of the linearisation of channel kinetics (in this case with a single nonlinear gating variable) responsible for subthreshold oscillatory behaviour around the steady state [6, 9–11]. The constants $D_i$ and $\tau _i$ in Eq. (1) can be found in terms of the electrical parameters of the cell membrane as $D_i=a_i/(4R_{a,i} C_i)$ and $\tau _i=C_iR_i$, where $a_i$ is the diameter and $R_{a,i}$ is the specific cytoplasmic resistivity of branch *i*. The term $I_{\mathrm{inj},i}(x,t)$ models an external current applied to this branch. The dendritic structure of each cell is attached to an equipotential soma of the diameter $a_\mathrm{S}$ modelled by the ‘LRC’ circuit with the parameters $C_\mathrm{S}=C_\mathrm{soma} \pi a^2_\mathrm{S}$, $R_\mathrm{S}=R_\mathrm{soma}/(\pi a^2_\mathrm{S})$, $L_\mathrm{S}=L_\mathrm{soma}/(\pi a^2_\mathrm{S})$ and $r_\mathrm{S}= r_\mathrm{soma}/(\pi a^2_\mathrm{S})$. Moreover, individual branches of different cells can be connected by gap junctions described by a conductance parameter $g_\mathrm{GJ}$.

Equations (1) and (2) for each dendritic segment must be accompanied by additional equations describing the dynamics of voltage at the two ends of a segment. If the proximal ($X=0$) or distal ($X={\widehat{\mathcal {L}}}_i$) end of a branch is a branching node point, the continuity of the potential across a node and Kirchhoff’s law of conservation of current are imposed. For example, boundary conditions for a node shown in Fig. 1 take the form:

3 $$\begin{aligned}&V_j({\widehat{\mathcal {L}}}_j,t)=V_n(0,t)=V_k(0,t), \end{aligned}$$

4 $$\begin{aligned}&\frac{1}{r_{a,j}}\frac{\partial V_j}{\partial X} \bigg |_{X={\widehat{\mathcal {L}}}_j}=\frac{1}{r_{a,n}}\frac{\partial V_n}{\partial X} \bigg |_{X=0}+\frac{1}{r_{a,k}}\frac{\partial V_k}{\partial X} \bigg |_{X=0}, \end{aligned}$$

where $r_{a,j}=4R_{a,j}/(\pi a_j^2)$ is the axial resistance of branch *j*. If a branch terminates at $X={\widehat{\mathcal {L}}}_i$, we have either a no-flux (a closed-end) boundary condition

5 $$\begin{aligned} \frac{\partial V_i}{\partial X}\bigg |_{X={\widehat{\mathcal {L}}}_i}=0 , \end{aligned}$$

or a zero value (an open-end) boundary condition

6 $$\begin{aligned} V_i({\widehat{\mathcal {L}}}_i,t)=0. \end{aligned}$$

A lumped soma can be treated as a special node point with the somatic membrane voltage $V_\mathrm{S}(t)$ and the following set of equations which imposes special boundary conditions on the proximal ends of branches connected to the soma:

7 $$\begin{aligned} V_\mathrm{S}(t)= & {} V_j(0,t), \end{aligned}$$

8 $$\begin{aligned} C_\mathrm{S}\frac{\mathrm{d}V_\mathrm{S}}{\mathrm{d}t}= & {} -\frac{V_\mathrm{S}}{R_\mathrm{S}}+\sum _j \left. \frac{1}{r_{a,j}}\frac{\partial V_j}{\partial X}\right| _{X=0}-I_\mathrm{S}, \end{aligned}$$

9 $$\begin{aligned} L_\mathrm{S}\frac{\mathrm{d}I_\mathrm{S}}{\mathrm{d}t}= & {} -r_\mathrm{S}I_\mathrm{S}+V_\mathrm{S}, \end{aligned}$$

where the sum in Eq. (8) is over all branches connected to the soma. If the branches of two cells are coupled by a gap junction, the location of this coupling can be treated as a special node point on an extended branching structure. This gap-junctional (GJ) node requires the following set of boundary conditions (given here with an assumption that it is placed at $X=0$):

10 $$\begin{aligned} V_{m^-}(0,t)=V_{m^+}(0,t),\qquad V_{n^-}(0,t)=V_{n^+}(0,t) , \end{aligned}$$

and

11 $$\begin{aligned}&\frac{1}{r_{a,m}}\left( \frac{\partial V_{m^-}}{\partial X}\bigg |_{X=0} +\frac{\partial V_{m^+}}{\partial X}\bigg |_{X=0}\right) \nonumber \\&=g_\mathrm{GJ}(V_{m^-}(0,t)-V_{n^-}(0,t)), \end{aligned}$$

12 $$\begin{aligned}&\frac{1}{r_{a,n}}\left( \frac{\partial V_{n^-}}{\partial X}\bigg |_{X=0} +\frac{\partial V_{n^+}}{\partial X}\bigg |_{X=0}\right) \nonumber \\&=g_\mathrm{GJ}(V_{n^-}(0,t)-V_{m^-}(0,t)), \end{aligned}$$

where $$m^{-}$$ and $$m^{+}$$ ($$n^{-}$$ and $$n^{+})$$ are two segments of branch *m* (branch *n*) on the left and right from a gap junction (see Fig. 1), respectively. The expressions in (10) reflect continuity of the potential across individual branches *m* and *n*, and Eqs. (11) and (12) enforce conservation of current.

The whole network in Fig. 1 can be viewed as a graph structure (which can be cyclic) with different types of nodes: a terminal, a regular branching node, a somatic node or the GJ node. The voltage dynamics along the network structure are described by linear equations, and therefore, the model’s behaviour can be studied by constructing the network response function known as the Green’s function, ${\widehat{G}}_{ij}(X,Y;t)$. This function describes the voltage response at the location *X* on branch *i* in response to a Dirac delta pulse applied to the location *Y* on branch *j* at time $t = 0$.

## Method of local point matching for finding the Green’s functions

Our method is based on and developed from the sum-over-trips approach for calculating the Green’s function on a network of electrically coupled neuronal cells [19]. Considering a network of cells as a single extended graph structure with labelled branches $$\{1, 2, \dots , i,\dots ,$$$$~k,\dots ,~j,\dots \}$$, the generalised sum-over-trips framework allows one to construct the Green’s function for the whole structure in the Laplace domain, $${G}_{ij}(X,Y;\omega )$$. After Laplace transforming Eqs. (1) and (2) with initial conditions $$V_i(X,0)=0$$ and $$I_i(X,0)=0$$, we obtain an ordinary differential equation for each branch *i*:

13 $$\begin{aligned} -\frac{\mathrm{d}^2V_i(X,\omega )}{\mathrm{d}X^2}+\gamma _i^2(\omega )V_i(X,\omega )=\frac{I_{\mathrm{inj},i}(X,\omega )}{C_i D_i}, \end{aligned}$$

where

14 $$\begin{aligned} \gamma _i^2(\omega )=[\tau _i^{-1}+\omega +(C_i(r_i+\omega L_i))^{-1}]/D_i. \end{aligned}$$

**Fig. 2:**
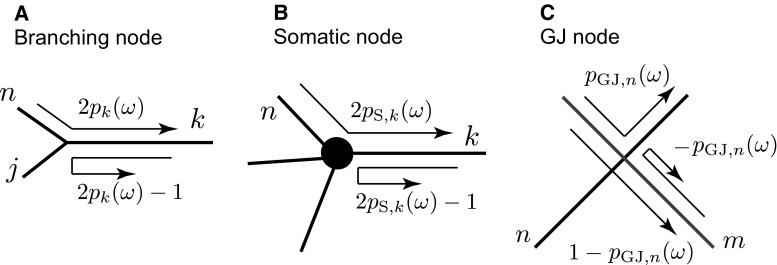
Sum-over-trip rules for different types of nodes
- *Somatic node*: $A_\mathrm{trip}(\omega )$ is multiplied by a factor $2p_{\mathrm{S},k}(\omega )$ or $2p_{\mathrm{S},k}(\omega )-1$ (see Fig. 2b), where
  17 $$\begin{aligned} p_{\mathrm{S},k}(\omega )= & {} \frac{z_k(\omega )}{\sum _n z_n(\omega )+z_\mathrm{S}(\omega )}, \end{aligned}$$
  18 $$\begin{aligned} z_\mathrm{S}(\omega )= & {} C_\mathrm{S}\omega +R_\mathrm{S}^{-1}+(r_\mathrm{S}+L_\mathrm{S}\omega )^{-1}. \end{aligned}$$
- *GJ node*: $A_\mathrm{trip}(\omega )$ is multiplied by a factor $p_{\mathrm{GJ},n}(\omega )$, $1-p_{\mathrm{GJ},n}(\omega )$ or $-p_{\mathrm{GJ},n}(\omega )$ (see Fig. 2c), where
  19 $$\begin{aligned} p_{\mathrm{GJ},n}(\omega )=\frac{z_n(\omega )}{z_m(\omega )+z_n(\omega )+2R_\mathrm{GJ}z_m(\omega )z_n(\omega )} \end{aligned}$$
  and $$R_\mathrm{GJ}=1/g_\mathrm{GJ}$$.
- *Terminal*: $A_\mathrm{trip}(\omega )$ is multiplied by $+1$ for the closed-end boundary or by $-1$ for the open-end boundary condition.

Considering an injected current in the form of a Dirac delta pulse and rescaling each branch *k* of the network by its own characteristic function $\gamma _k(\omega )$ as ${\mathcal {L}}_{k}=\gamma _{k}(\omega ){\widehat{\mathcal {L}}}_k$, it is possible to derive (see [6, 19]) that the Green’s function on a scaled network ($x=\gamma _{i}(\omega )X$, $y=\gamma _{j}(\omega )Y$) takes the form of an infinite series expansion

15 $$\begin{aligned} {G}_{ij}(x,y;\omega )=\frac{1}{2D_j\gamma _j(\omega )}\sum _\mathrm{{trips}}A_\mathrm{{trip}}(\omega )f(L_\mathrm{{trip}}(x,y;\omega )), \end{aligned}$$

where $f(x)=\mathrm{e}^{-|x|}$ and $L_\mathrm{{trip}}(x,y;\omega )$ is the length of a trip along the network structure that starts at the point $x=\gamma _i(\omega )X$ on branch *i* and ends at the point $y=\gamma _j(\omega )Y$ on branch *j*. The trips coefficients $A_{\mathrm{trip}}(\omega )$ in (15) are chosen according to the following set of rules:

- Initiate $$A_\mathrm{trip}(\omega )=1$$.
- *Branching node*: $A_\mathrm{trip}(\omega )$ is multiplied by a factor $2p_k(\omega )$ or $2p_k(\omega )-1$ (see Fig. 2a), where $p_k(\omega )$ is a branch factor defined by
  16 $$\begin{aligned} p_k(\omega )=\frac{z_k(\omega )}{\sum _n z_n(\omega )},\qquad z_k(\omega )=\frac{\gamma _k(\omega )}{r_{a,k}}. \end{aligned}$$

We refer the reader to [19] for a detailed summary of the generalised sum-over-trip method and the trip coefficients.

**Fig. 3:**
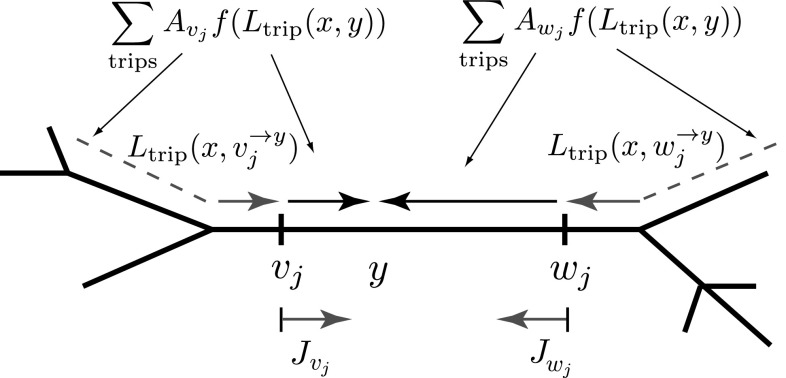
Construction of $J_{y}$ by dividing the trips into two classes represented by the functions $J_{v_j}$ and $J_{w_j}$. *Dashed lines* indicate all possible trips on a network

**Fig. 4:**
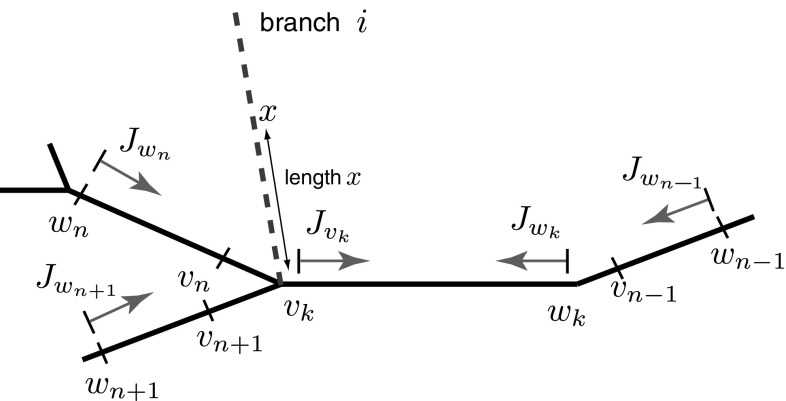
Part of a network with the placed pairs of points $(v_k,w_k)$ and the corresponding functions $J_{v_k}$ and $J_{w_k}$

Next, we provide a description of the main steps behind the derivation of the new method of local point matching together with the algorithmic summary of this method, the detailed derivation of which can be found in “Appendix 1”. Note that $\omega$ is omitted for compactness from this point. All trips terminated at point *y* can be divided into two classes separated by the direction of the last part of the trip. Placing two points $v_j$ and $w_j$ on segment *j* as shown in Fig. 3, we consider one class which includes the trips with $L_\mathrm{trip}(x,v_j^{\rightarrow y})$ approaching *y* from the left (named as $J_{v_j}$) and the other class which includes the trips with $L_\mathrm{trip}(x,w_j^{\rightarrow y})$ approaching *y* from the right (named as $J_{w_j}$). Without constructing the actual trips, it is possible to show that all trips ending at *y*, named as $J_y$ and from (15) having the form

20 $$\begin{aligned} J_{y}=\sum _\mathrm{trips}A_\mathrm{trip}f(L_\mathrm{trip}(x,y)), \end{aligned}$$

can then be found as a linear combination of the unknown functions $J_{v_j}$ and $J_{w_j}$ belonging to these two classes. Likewise, we can partition trips on all other branches by placing a pair of points $(v_k,w_k)$ on each segment *k* and introducing two classes of trips $J_{v_k}$ and $J_{w_k}$ (see Fig. 4). Each unknown function $J_{v_k}$ can then be written as a linear combination of the nearest unknown functions $J_{w_n}$, $J_{w_{n+1}}$ and $J_{w_k}$ which are heading towards point $v_k$. Similarly, the unknown function $J_{w_k}$ can be written as a linear combination of the nearest unknown functions $J_{v_k}$ and $J_{w_{n-1}}$ heading towards point $w_k$. This leads to a linear system of 2*N* algebraic equations for all unknown functions $J_{v_k}$ and $J_{w_k}$ defined on each segment $k=1,\dots ,N$, where *N* is the number of dendritic segments in the network. Solving this linear system for $J_{v_k}$ and $J_{w_k}$, we can then find the unknown function $J_y$ and, as a result, the Green’s function $G_{ij}(x,y;\omega )$.

*Summary of method*

Here we summarise the main steps of an algorithm for constructing the compact Green’s functions in the Laplace domain for an arbitrary neuronal network and refer the reader to “Appendix 1” for the detailed derivation of this method.

1. The physical length ${\widehat{{\mathcal {L}}}}_{k}$ of each branch *k* is scaled by its own characteristic function $\gamma _{k}(\omega )$ given by Eq. (14).
2. Place a pair of points $(v_k,w_k)$ on each segment *k* (see Fig.  4). Assume that $v_k$ and $w_k$ are placed infinitesimally close to both ends of the branch. Trips from $v_k$ and $w_k$ can move only towards each other (see red vectors in Fig.  4). Construct a system of linear algebraic equations for all $J_{v_k}$ and $J_{w_k}$. For example, the function $J_{v_k}$ in Fig. 4 depends on a linear combination of the terms with $J_{w_n}$, $J_{w_k}$ and $J_{w_{n+1}}$ (if the branch *i* with point *x* is absent; otherwise, an additional term $a_{ik}f(x)$ must be included in the linear combination, where $a_{ik}$ is a coefficient for a trip passing from segment *i* to segment *k*). The function $J_{w_k}$ in Fig. 4 depends on a linear combination of the terms with $J_{v_k}$ and $J_{w_{n-1}}$. The constructed linear combinations for the unknown functions $J_{v_k}$ and $J_{w_k}$ include trip coefficients $a_{nk}$ for trips passing from segment *n* to segment *k* and trip coefficients $a_{kk}$ for trips reflecting at the end points of segment *k*. These coefficients are obtained from the sum-over-trips rules summarised in Fig. 2.
3. Solve the constructed linear system of algebraic equations and therefore find $J_{v_j}$ and $J_{w_j}$ for a pair of points $(v_j,w_j)$ placed on segment *j* which includes point *y*, ($0<y<{\mathcal {L}}_j$), see Fig. 3.
4. Find the function $J_y$ as $J_y=f(y)J_{v_j}+f({\mathcal {L}}_j-y)J_{w_j}$ or, if *x* is located on branch *j*, using $J_y=f(y)J_{v_j}+f({\mathcal {L}}_j-y)J_{w_j}+f(x-y)$.
5. Find $$G_{ij}(x,y)$$ as $$G_{ij}(x,y)=J_y/(2D_j\gamma _j)$$.
6. Rescale the coordinates $X=x/\gamma _i(\omega )$ and $Y=y/\gamma _j(\omega )$ and take the inverse Laplace transform (InvLT) of $G_{ij}(X,Y;\omega )$ to obtain the Green’s function ${\widehat{G}}_{ij}(X,Y;t)$.

If point *y* is located at a node (i.e. $y=0$ or $y={\mathcal {L}}_j$), due to the continuity of the potential at the boundaries the method can be easily applied by initially, assuming that *y* is placed on segment *j* slightly away from this node and, after the Green’s function is constructed, considering that $y=0$ or $y={\mathcal {L}}_j$. A similar approach can be used if point *x* is also located at one of the nodes.

Note that spatially extended neurons coupled by gap junctions into an arbitrary neuronal network might develop a graph structure with cycles, and our method of local point matching (as well as the original sum-over-trips method) can support such structures.

## Applications

### A soma and dendrite model

**Fig. 5:**
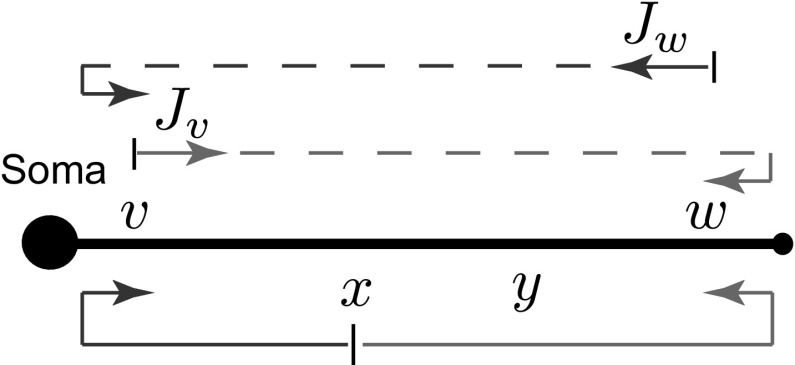
A soma and dendrite model. Terms of Eq.  (21) are shown by *blue arrows*, and terms of Eq.  (22) are shown by *red arrows*

Here we consider a simple model of a dendrite with a lumped soma attached to it at $x=0$ (see Fig. 5). We assume that a length of the dendrite, ${\widehat{{\mathcal {L}}}}$, is scaled by its characteristic function $\gamma (\omega )$, i.e. ${\mathcal {L}}=\gamma (\omega ){\widehat{{\mathcal {L}}}}$ . If the dendrite is terminated with a closed-end boundary condition (i.e. has a factor $+1$ at the terminal), a system of linear equations for $J_{v}$ and $J_{w}$ corresponding to a pair of points (*v*, *w*) takes the following form

21 $$\begin{aligned} J_{v}= & {} J_{w}f({\mathcal {L}})(2p_\mathrm{S}-1)+ f(x)(2p_\mathrm{S}-1), \end{aligned}$$

22 $$\begin{aligned} J_{w}= & {} J_{v}f({\mathcal {L}}) + f({\mathcal {L}}-x), \end{aligned}$$

where $p_\mathrm{S}$ can be found from (17) and (18) as

23 $$\begin{aligned} p_\mathrm{S}=\frac{\gamma (\omega )/r_{a}}{\gamma (\omega )/r_{a}+C_\mathrm{S}\omega +R_\mathrm{S}^{-1}+(r_\mathrm{S}+L_\mathrm{S}\omega )^{-1}}. \end{aligned}$$

Solving the system, we can find that

24 $$\begin{aligned} J_v= & {} \frac{(2p_\mathrm{S}-1)[f(2{\mathcal {L}}-x)+f(x)]}{1-(2p_\mathrm{S}-1)f(2{\mathcal {L}})}, \end{aligned}$$

25 $$\begin{aligned} J_w= & {} \frac{(2p_\mathrm{S}-1)f({\mathcal {L}}+x)+f({\mathcal {L}}-x)}{1-(2p_\mathrm{S}-1)f(2{\mathcal {L}})}, \end{aligned}$$

and then obtain $J_{y}$ as

26 $$\begin{aligned} J_y=J_vf(y)+J_wf({\mathcal {L}}-y)+f(x-y), \end{aligned}$$

and finally, the Green’s function in the Laplace domain

27 $$\begin{aligned} G(x,y)=\frac{J_y}{2D\gamma }. \end{aligned}$$

This compact solution for the Green’s function is equivalent to a solution in the form of an infinite series expansion obtained by using the sum-over-trips method [18]:

28 $$\begin{aligned}&{G}(x,y)=\sum _{n=0}^{\infty }(2p_\mathrm{S}-1)^n[ f(y-x+2n{{\mathcal {L}}})\nonumber \\&\quad +\,(2p_\mathrm{S}\!-\!1)[f(y\!+\!x\!+\!2n{\mathcal {L}})\!+\!f(\!-\!(y\!-\!x) \!+\!2{\mathcal {L}}(n\!+\!1))] \nonumber \\&\quad +\,f(-(y+x)+2{\mathcal {L}}(n+1))]/(2D\gamma ). \end{aligned}$$

If the output is measured at the soma ($x=0$), the compact Green’s function takes the form

29 $$\begin{aligned} G(0,y)=\frac{p_\mathrm{S}[f(y)+f(2{\mathcal {L}}-y)]}{D\gamma [1-(2p_\mathrm{S}-1)f(2{\mathcal {L}})]}, \end{aligned}$$

and in the case of the somatic stimulation, it is simply

30 $$\begin{aligned} G(0,0)=\frac{p_\mathrm{S}[1+f(2{\mathcal {L}})]}{D\gamma [1-(2p_\mathrm{S}-1)f(2{\mathcal {L}})]}. \end{aligned}$$

**Fig. 6:**
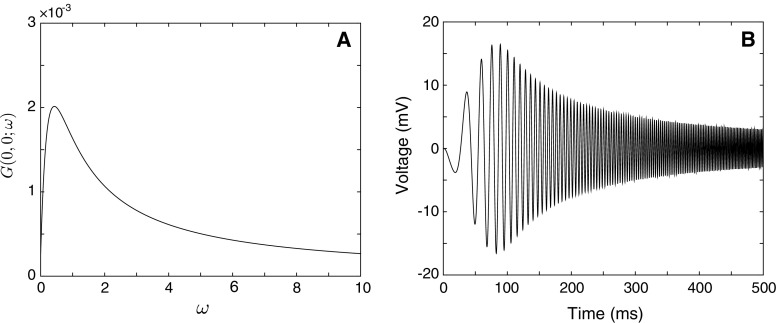
**a** Somatic Green’s function in the frequency domain given by (30). Dendritic parameters: ${\widehat{\mathcal {L}}}=50\,\mu {\mathrm{m}}$, $a=2\,\mu {\mathrm{m}}$, $C=1\,\mu {\mathrm{F}}\cdot \hbox {cm}^{-2}$, $R=2000\,\Omega \cdot \hbox {cm}^2$, $R_a=100\,\Omega \cdot \hbox {cm}$, $r=1000\,\Omega \cdot \hbox {cm}^2$, $L=5\,\mathrm{H}\cdot \hbox {cm}^2$. Somatic parameters: $a_\mathrm{S}=25\,\mu \mathrm{m}$, $C_\mathrm{soma}=1\,\mu {\mathrm{F}}\cdot \hbox {cm}^{-2}$, $R_\mathrm{soma}=2000\,\Omega \cdot \hbox {cm}^2$, $r_\mathrm{soma}=100\,\Omega \cdot \hbox {cm}^2$, $L_\mathrm{soma}=5\,\mathrm{H}\cdot \hbox {cm}^2$. **b** Somatic voltage profile in response to a stimulus $I_\mathrm{chirp}(t)$ with parameters $\omega _\mathrm{chirp}=0.003$, $A_\mathrm{chirp}=0.2\,\mathrm{nA}$

Figure 6a shows a profile of the somatic Green’s function given by (30). In Fig. 6b, we plot a somatic voltage profile in response to a chirp stimulus ${\widehat{I}}_\mathrm{chirp}(t) = A_\mathrm{chirp} \mathrm{sin}(\omega _\mathrm{chirp}t^{2})$, found as $V(t)=\mathrm{InvLT}[G(0,0)I_\mathrm{chirp}(\omega )]$, where $I_\mathrm{chirp}(\omega )$ is the Laplace transform of ${\widehat{I}}_\mathrm{chirp}(t)$.

The model can be easily modified for the case of a semi-infinite dendrite. Assuming ${\mathcal {L}}\rightarrow \infty$, we have $f({\mathcal {L}})\rightarrow 0$ and from Eqs. (24) and (25)

31 $$\begin{aligned} J_v= & {} (2p_\mathrm{S}-1) f(x), \end{aligned}$$

32 $$\begin{aligned} J_w= & {} 0. \end{aligned}$$

Then $J_{y}$ in (27) takes the form

33 $$\begin{aligned} J_y=(2p_\mathrm{S}-1)f(x+y)+f(x-y), \end{aligned}$$

and the somatic Green’s function simply becomes

34 $$\begin{aligned} G(0,y)=\frac{p_\mathrm{S}f(y)}{D\gamma }. \end{aligned}$$

**Fig. 7:**
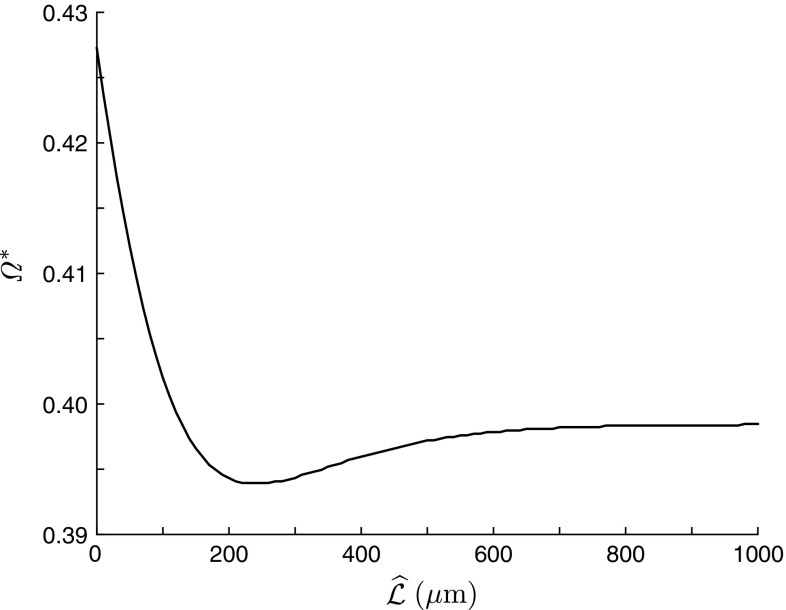
Preferred frequency ${\varOmega }^*$ as a function of the dendritic length ${\widehat{{\mathcal {L}}}}$. Other parameters as in Fig. 6

Resonant dynamics of the model can be characterised by a preferred frequency ${\varOmega }^*$ at which the Green’s function has its maximum. Figure 6b clearly shows resonant behaviour of the system maximising the voltage response for particular frequencies. In Fig. 7, we plot a preferred frequency as a function of a dendritic length ${\widehat{{\mathcal {L}}}}$ when *x* and *y* are placed at the soma. This dependence is obtained as a solution of the implicit equation

35 $$\begin{aligned} \frac{\partial G(0,0;\omega )}{\partial \omega }=0, \qquad \omega \ge 0. \end{aligned}$$

The plot demonstrates a nonmonotonic trend with a minimal value within a realistic range of dendritic lengths indicating a considerable effect of dendritic extents on the model’s dynamics.

### A two-cell simplified network

Here we demonstrate how our method can be applied to a two-cell network of either identical or nonidentical cells coupled by a dendro-dendritic gap junction. In each case, we obtain the compact solutions for the Green’s functions, Eqs. (40)–(43) for the two-cell identical network and Eqs. (49)–(56) for the two-cell nonidentical network, which can inform us about the roles of individual parameters on the network dynamics.

**Fig. 8:**
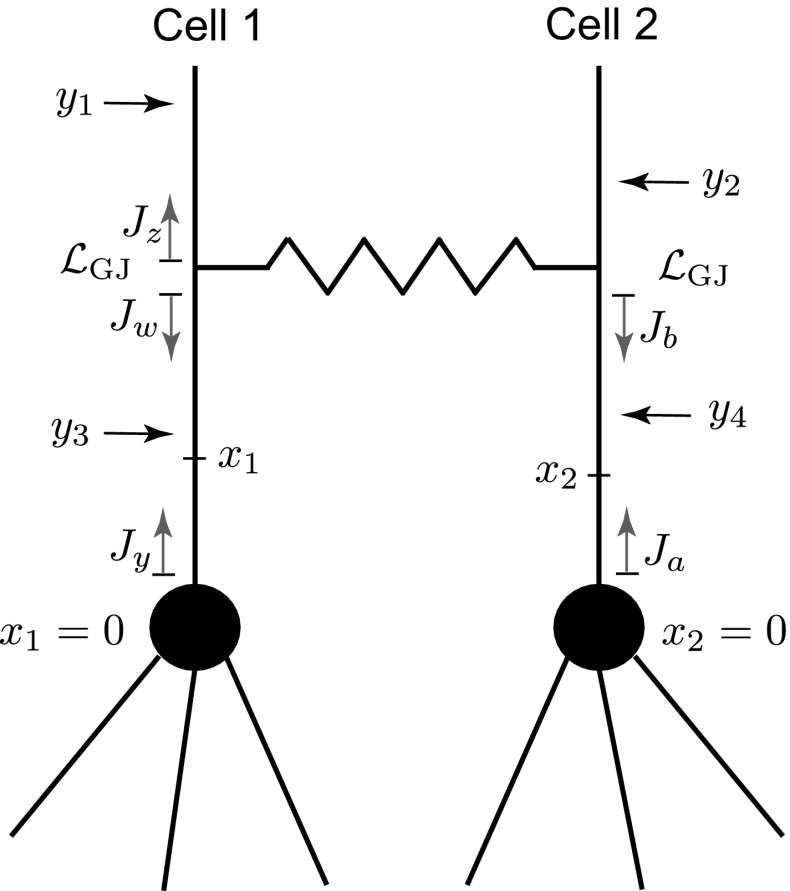
A schematic of a two-cell simplified network

We start by considering a model of two identical cells, either of which consists of a soma and *N* attached semi-infinite dendrites as shown in Fig. 8. We assume that the biophysical properties of all dendritic segments are the same and that the physical lengths are scaled by the characteristic function $\gamma (\omega )$ given by (14). The gap junction is located at some distance ${\mathcal {L}}_\mathrm{GJ}$ away from the cell bodies. We assume that this network can receive stimuli in four different locations mimicking distal ($y_{1}$ and $y_{2}$) and proximal ($y_{3}$ and $y_{4}$) inputs. Points of output $x_{1}$ (for Cell 1) and $x_{2}$ (for Cell 2) are located between either soma and the gap junction.

Using our method, we can construct a linear system of algebraic equations for the functions $J_{a}, J_{b}, J_{y}$ and $J_{w}$ in the case of placing output at $x_2$ (see Fig. 8):

36 $$\begin{aligned} J_a= & {} J_b f({\mathcal {L}}_\mathrm{GJ}) (2p_\mathrm{S}-1)+f(x_2)(2p_\mathrm{S}-1), \end{aligned}$$

37 $$\begin{aligned} J_b= & {} J_y f({\mathcal {L}}_\mathrm{GJ}) p_\mathrm{GJ}+J_a f({\mathcal {L}}_\mathrm{GJ})(-p_\mathrm{GJ})\nonumber \\&+\,f({\mathcal {L}}_\mathrm{GJ}-x_2)(-p_\mathrm{GJ}), \end{aligned}$$

38 $$\begin{aligned} J_y= & {} J_w f({\mathcal {L}}_\mathrm{GJ}) (2p_\mathrm{S}-1), \end{aligned}$$

39 $$\begin{aligned} J_w= & {} J_y f({\mathcal {L}}_\mathrm{GJ}) (-p_\mathrm{GJ})+J_a f({\mathcal {L}}_\mathrm{GJ})p_\mathrm{GJ}\nonumber \\&+\,f({\mathcal {L}}_\mathrm{GJ}-x_2)p_\mathrm{GJ}. \end{aligned}$$

This system of equations can be easily solved analytically (see “Appendix 2”). The Green’s functions for four individual inputs for Cell 2 can then be found as

40 $$\begin{aligned} G_{2}(x_2,y_1)= & {} \frac{1}{2D\gamma }\frac{p_\mathrm{GJ}+p_\mathrm{GJ}\alpha }{q}{{\widetilde{F}}}(x_2,y_1), \end{aligned}$$

41 $$\begin{aligned} G_{2}(x_2,y_2)= & {} \frac{1}{2D\gamma }\frac{1-p_\mathrm{GJ}+p_\mathrm{GJ}\alpha }{q}{{\widetilde{F}}}(x_2,y_2), \end{aligned}$$

42 $$\begin{aligned} G_{2}(x_2,y_3)= & {} \frac{1}{2D\gamma }\frac{p_\mathrm{GJ}f(2{\mathcal {L}}_\mathrm{GJ})}{q}{{\widetilde{F}}}(x_2,0){{\widetilde{F}}}(y_3,0), \end{aligned}$$

43 $$\begin{aligned} G_{2}(x_2,y_4)= & {} \frac{1}{2D\gamma }\bigg [f(x_2+y_4)(2p_\mathrm{S}-1)+f(x_2-y_4)\nonumber \\&-\,\frac{p_\mathrm{GJ}f(2{\mathcal {L}}_\mathrm{GJ})}{q}{\widetilde{F}}(x_2,0){\widetilde{F}}(y_4,0)\bigg ], \end{aligned}$$

where

44 $$\begin{aligned} \alpha= & {} (2p_\mathrm{S}-1)f(2{\mathcal {L}}_\mathrm{GJ}), \end{aligned}$$

45 $$\begin{aligned} q= & {} 1+2p_\mathrm{GJ}\alpha , \end{aligned}$$

and

46 $$\begin{aligned} {\widetilde{F}}({\mathrm{m}},\mathrm{n})=f(\mathrm{m+n})(2{p_S}-1)+\frac{f(\mathrm{n})}{f({\mathrm{m}})}. \end{aligned}$$

Here $p_\mathrm{S}$ and $p_\mathrm{GJ}$ can be found from Eqs. (17) and (19), respectively. As the cells are identical and due to the symmetry of the input locations, the corresponding Green’s functions for Cell 1 can be easily obtained from Eqs. (40)–(43).

**Fig. 9:**
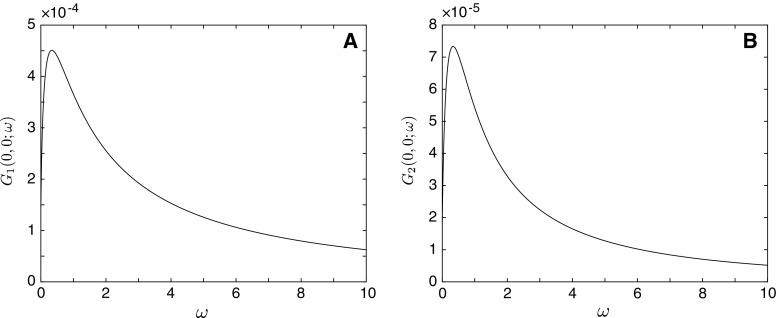
**a** Somatic Green’s function in the Laplace domain for Cell 1 when input is placed at $y_{3}=0$. **b** Somatic Green’s function in the Laplace domain for Cell 2 when input is placed at $y_{3}=0$. Biophysical parameters of the cells’ membrane as in Fig. 6. Gap-junctional parameters: ${\mathcal {L}}_\mathrm{GJ}=50\,\mu \mathrm{m}$, $R_\mathrm{GJ}=100\,\mathrm M{\varOmega }$

In Fig. 9, we plot the Green’s functions at the soma of each cell ($x_1=0$ and $x_2=0$) in response to a stimulus $y_{3}=0$ applied to Cell 1. Note that Eqs. (40) and (41) are equivalent to the solutions for the Green’s functions in the form of an infinite series expansion found using the ‘method of words’ in [19]. Truncated series solutions with the index of summation $n\ge 10$ match the compact Green’s functions obtained here (not shown).

Assuming that two distal inputs $y_1$ and $y_2$ are applied at equal distances from each soma ($y_1=y_2>{\mathcal {L}}_\mathrm{GJ}$), the Green’s function for each soma is identical:

47 $$\begin{aligned}&G_{1}(0,y_1)+G_{1}(0,y_2)=G_{2}(0,y_1)+G_{2}(0,y_2)\nonumber \\&\quad =\frac{{\widetilde{F}}(0,y_1)}{2D\gamma }=\frac{p_\mathrm{S} f(y_1)}{D\gamma }, \end{aligned}$$

Similarly, for the case of two proximal inputs $y_3$ and $y_4$ placed at the same distance away from each soma ($y_3=y_4<{\mathcal {L}}_\mathrm{GJ}$), the somatic Green’s function for each cell has the same form:

48 $$\begin{aligned} G_{1}(0,y_3)+G_{1}(0,y_4)= & {} G_{2}(0,y_3)+G_{2}(0,y_4)\nonumber \\= & {} \frac{{\widetilde{F}}(0,y_3)}{2D\gamma }=\frac{p_\mathrm{S} f(y_3)}{D\gamma }. \end{aligned}$$

Both solutions are independent of $g_\mathrm{GJ}$ and ${\mathcal {L}}_\mathrm{GJ}$ and have the form of Eq. (34) for the soma and dendrite model, i.e. without the presence of the gap junction.

Using our method, we can also construct analytical solutions for the Green’s functions for a network of two nonidentical cells. The response functions at the soma of Cell 2 take the forms

49 $$\begin{aligned} G_{2}(0,y_1)= & {} \frac{p_\mathrm{S_2}}{D_1\gamma _1}f({\mathcal {L}}_2+y_1-{\mathcal {L}}_1)\frac{p_\mathrm{GJ,1}+p_\mathrm{GJ,1}\alpha _1}{q}, \nonumber \\ \end{aligned}$$

50 $$\begin{aligned} G_{2}(0,y_2)= & {} \frac{p_\mathrm{S_2}}{D_2\gamma _2}f(y_2)\frac{1-p_\mathrm{GJ,1}+p_\mathrm{GJ,2}\alpha _1}{q}, \end{aligned}$$

51 $$\begin{aligned} G_{2}(0,y_3)= & {} \frac{p_\mathrm{S_2}}{D_1\gamma _1}\frac{p_\mathrm{GJ,1}}{q}{\widetilde{F}}_1(y_3,{\mathcal {L}}_1+{\mathcal {L}}_2), \end{aligned}$$

52 $$\begin{aligned} G_{2}(0,y_4)= & {} \frac{p_\mathrm{S_2}}{D_2\gamma _2}\bigg [f(y_4)-\frac{p_\mathrm{GJ,1}}{q} {\widetilde{F}}_2(y_4,2{\mathcal {L}}_2)\bigg ], \end{aligned}$$

and using symmetry, the response functions at the soma of Cell 1 can be found as

53 $$\begin{aligned} G_{1}(0,y_2)= & {} \frac{p_\mathrm{S_1}}{D_2\gamma _2}f({\mathcal {L}}_1+y_2-{\mathcal {L}}_2)\frac{p_\mathrm{GJ,2}+p_\mathrm{GJ,2}\alpha _2}{q}, \nonumber \\ \end{aligned}$$

54 $$\begin{aligned} G_{1}(0,y_1)= & {} \frac{p_\mathrm{S_1}}{D_1\gamma _1}f(y_1)\frac{1-p_\mathrm{GJ,2}+p_\mathrm{GJ,1}\alpha _2}{q}, \end{aligned}$$

55 $$\begin{aligned} G_{1}(0,y_4)= & {} \frac{p_\mathrm{S_1}}{D_2\gamma _2}\frac{p_\mathrm{GJ,2}}{q}{\widetilde{F}}_2(y_4,{\mathcal {L}}_1+{\mathcal {L}}_2),\end{aligned}$$

56 $$\begin{aligned} G_{1}(0,y_3)= & {} \frac{p_\mathrm{S_1}}{D_1\gamma _1}\bigg [ f(y_3)-\frac{p_\mathrm{GJ,2}}{q} {\widetilde{F}}_1(y_3,2{\mathcal {L}}_1)\bigg ], \end{aligned}$$

where, for $$k=1, 2$$,

57 $$\begin{aligned}&\alpha _k=(2p_{\mathrm{S}_k}-1)f(2{\mathcal {L}}_k),\end{aligned}$$

58 $$\begin{aligned}&q=1+p_\mathrm{GJ,2}\alpha _1+p_\mathrm{GJ,1}\alpha _2,\end{aligned}$$

59 $$\begin{aligned}&{\widetilde{F}}_k({\mathrm{m}},\mathrm{n})=f({\mathrm{m}}+\mathrm{n})(2p_{\mathrm{S}_k}-1)+\frac{f(\mathrm{n})}{f({\mathrm{m}})},\end{aligned}$$

60 $$\begin{aligned}&p_{\mathrm{S}_{k}}=\frac{\gamma _{k}/r_{a,k}}{N\gamma _{k}/r_{a,k}+C_{\mathrm{S}_{k}}\omega +R_{\mathrm{S}_{k}}^{-1}+(r_{\mathrm{S}_{k}}+L_{\mathrm{S}_{k}}\omega )^{-1}},\nonumber \\ \end{aligned}$$

$\gamma _{k}=\gamma _{k}(\omega )$ is the characteristic function of the membrane of Cell *k*, ${\mathcal {L}}_k$ is the distance between the gap junction and the soma of Cell *k*, and $p_{\mathrm{GJ},k}$ is given by Eq. (19).

**Fig. 10:**
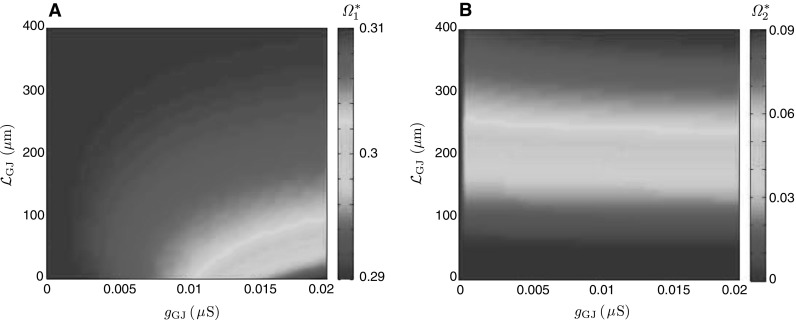
Preferred frequencies ${\varOmega }^*_1$ and ${\varOmega }^*_2$ at the soma of Cell 1 (**a**) and of Cell 2 (**b**). Dendritic parameters of Cell 1 as in Fig.  9, except $r_1=100\,{\Omega }\cdot \hbox {cm}^2$. The dendritic parameters of Cell 2: $a_2=0.4\,\mu \mathrm{m}$, $C_2=1\,\mu {\mathrm{F}}\cdot \hbox {cm}^{-2}$, $R_2=20{,}000\,\Omega \cdot \hbox {cm}^2$, $R_{a,2}=150\,\Omega \cdot \hbox {cm}$, and $r_2\rightarrow \infty$ (i.e. passive dendritic membrane). Both somas are passive

Using Eqs. (49)–(56), we can investigate how the strength and location of the gap junction affect the dynamics of the two-cell model. Here, we consider that a stimulus is applied to the soma of Cell 1 and construct a map

61 $$\begin{aligned} {\varPsi }: ({\mathcal {L}}_\mathrm{GJ}, g_\mathrm{GJ}) \rightarrow ({\varOmega }^*_1, {\varOmega }^*_2) \end{aligned}$$

for the preferred frequencies ${\varOmega }^*_1$ and ${\varOmega }^*_2$ at the soma of Cell 1 and Cell 2, respectively. This map is shown in Fig. 10. In this case, Cell 2 is assumed to be purely passive, and Cell 1 has a passive soma with resonant dendrites. The map indicates that the location of a gap junction plays a significant role in the dynamics of the network, unless the coupling is weak. Moreover, the initially passive soma of Cell 2 starts to demonstrate a resonant behaviour imposed by Cell 1 even for weak coupling.

**Fig. 11:**
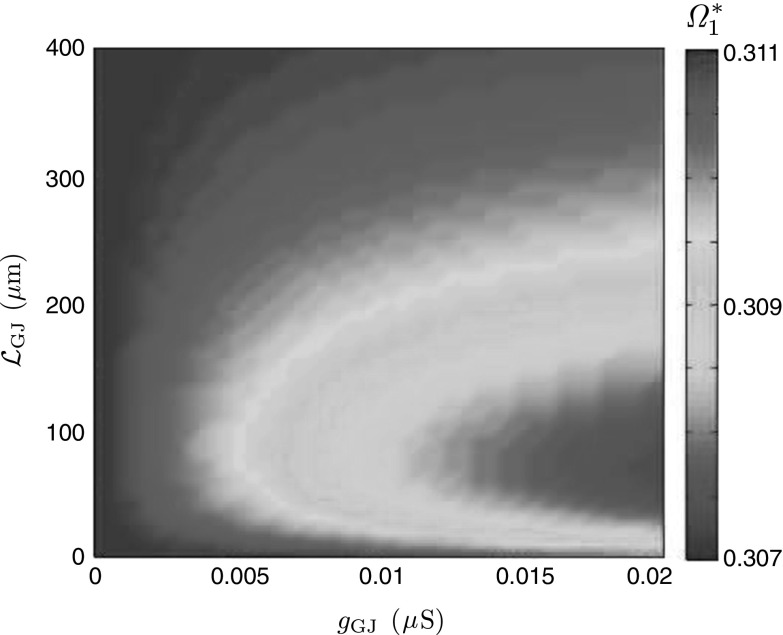
Preferred frequency ${\varOmega }^*_1$ at Soma 1. All parameters are the same as in Fig. 10, except $r_2=300\,\Omega \cdot \hbox {cm}^2$

Often it is difficult to measure experimentally locations and strengths of gap junctions in real neuronal networks. Knowledge of the inverse map

62 $$\begin{aligned} {\varPsi }^{-1}:({\varOmega }^*_1, {\varOmega }^*_2)\rightarrow ({\mathcal {L}}_\mathrm{GJ}, g_\mathrm{GJ}) \end{aligned}$$

from a pair of preferred frequencies (obtained from somatic sub-threshold stimulations) to $({\mathcal {L}}_\mathrm{GJ}, g_\mathrm{GJ})$ might provide estimates for gap-junctional parameters. However, the map ${\varPsi }$ is neither surjective nor injective (see, for example, Fig. 11 for a network of two resonant cells showing that the system may demonstrate the same resonant behaviour for two different gap-junctional locations, proximal and distal, and identical coupling strengths) making it mathematically impractical to obtain ${\varPsi }^{-1}$. At the same time, if a constraint on locations of gap junctions is imposed (e.g. proximal or distal), this may lead to a one-to-one correspondence between $({\mathcal {L}}_\mathrm{GJ}, g_\mathrm{GJ})$ and $({\varOmega }^*_1, {\varOmega }^*_2)$ and therefore assists in the estimation of gap-junctional parameters just from the somatic stimulations.

### A two-cell tufted network

**Fig. 12:**
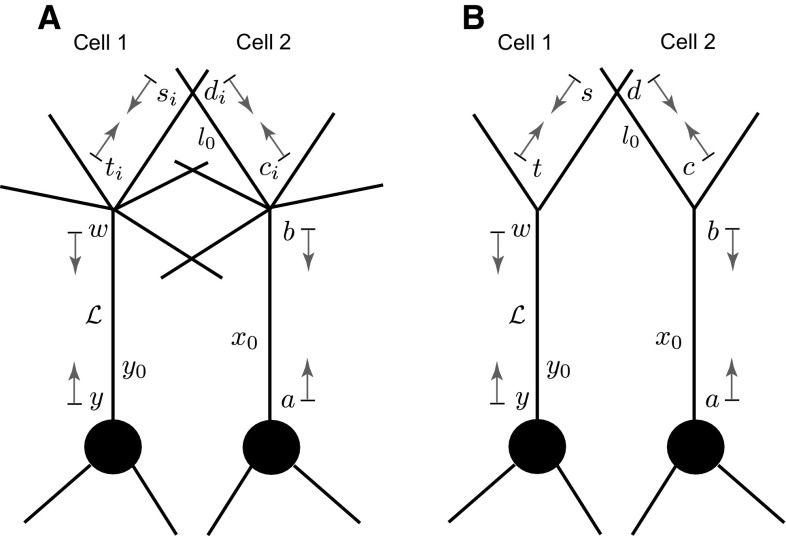
**a** A full two-cell tufted network model. **b** An equivalent reduced model

Now we consider a more realistic neuronal network consisting of two identical tufted or mitral cells. Each neuron has a soma attached to *N* dendritic branches, one of which is the primary dendrite with the tuft spanning from its end. Two cells are coupled in their tufts by dendro-dendritic gap junctions (see Fig. 12a). As in the previous model, we assume that the biophysical properties of all dendritic segments are the same and that the physical lengths are scaled by the characteristic function $\gamma (\omega )$. We consider that each cell has $n_\mathrm{T}$ segments in its tuft, and $n_\mathrm{GJ}$ of them possess identical single gap-junctional points located $l_{0}$ away from the end of the primary dendrite. The primary dendrite of each cell has the length ${\mathcal {L}}$, while the other branches are semi-infinite. For simplicity, we consider that the membrane of both cells is purely passive (i.e. $\gamma ^2(\omega )=(\tau ^{-1}+\omega )/D$); however, it is straightforward to generalise it for the resonant case.

Although it is possible to use our method and construct the compact Green’s functions for this tufted network, we will first demonstrate that there exists an equivalent reduced model with the simplified structure shown in Fig. 12b for which the compact solutions will then be constructed. Here we consider a reduction in the full model when external inputs cannot apply to any of the tufted dendrites. Notating the reduced network with the symbol $'$ we constrain the reduced model to have

63 $$\begin{aligned} {\mathcal {L}}'= & {} {\mathcal {L}}, \end{aligned}$$

64 $$\begin{aligned} l_0'= & {} l_0, \end{aligned}$$

65 $$\begin{aligned} p_\mathrm{S}'= & {} p_\mathrm{S}, \end{aligned}$$

66 $$\begin{aligned} p_\mathrm{D}'= & {} p_\mathrm{D}, \end{aligned}$$

where $p_\mathrm{S}$ constitutes part of the definition of a trip coefficient for the somatic node (see Fig. 2b), and $p_\mathrm{D}$ is a branch factor of the primary dendrite defined as in (16) and constitutes part of the definition of a trip coefficient for the branching node (see Fig. 2a). Equations (63) and (64) force the length of the primary dendrite and the location of a single gap junction in the reduced model to be the same as in the full tufted model. Placing $y_{0}$ on the primary dendrite of Cell 1, both models are equivalent if

67 $$\begin{aligned} J_{y_0}'=J_{y_0}. \end{aligned}$$

Using our method of local point matching, we can write down a system of algebraic equations for the full tufted model:

68 $$\begin{aligned} J_a= & {} J_bf({\mathcal {L}})(2p_\mathrm{S}-1)+f(x_0)(2p_\mathrm{S}-1), \end{aligned}$$

69 $$\begin{aligned} J_b= & {} \sum _{i} J_{d_i}f(l_0)2p_\mathrm{D}+J_af({\mathcal {L}})(2p_\mathrm{D}-1)\nonumber \\&+\,f({\mathcal {L}}-x_0)(2p_\mathrm{D}-1), \end{aligned}$$

70 $$\begin{aligned} J_{c_i}= & {} J_af({\mathcal {L}})2p_\mathrm{T}+J_{d_i}f(l_0)(2p_\mathrm{T}-1)\nonumber \\&+\,\sum _{j\ne i}J_{d_j}f(l_0)2p_\mathrm{T}+f({\mathcal {L}})2p_\mathrm{T}, \end{aligned}$$

71 $$\begin{aligned} J_{d_i}= & {} J_{c_i}f(l_0)(-p_\mathrm{GJ})+J_{t_i}f(l_0)p_\mathrm{GJ},\end{aligned}$$

72 $$\begin{aligned} J_{s_i}= & {} J_{c_i}f(l_0)p_\mathrm{GJ}+J_{t_i}f(l_0)(-p_\mathrm{GJ}),\end{aligned}$$

73 $$\begin{aligned} J_{t_i}= & {} J_yf({\mathcal {L}})2p_\mathrm{T}+J_{s_i}f(l_0)(2p_\mathrm{T}-1)\nonumber \\&+\,\sum _{j\ne i}J_{s_j}f(l_0)2p_\mathrm{T}, \end{aligned}$$

74 $$\begin{aligned} J_w= & {} \sum _{i} J_{s_i}f(l_0)2p_\mathrm{D}+J_yf({\mathcal {L}})(2p_\mathrm{D}-1), \end{aligned}$$

75 $$\begin{aligned} J_y= & {} J_wf({\mathcal {L}})(2p_\mathrm{S}-1),\end{aligned}$$

76 $$\begin{aligned} J_{y_0}= & {} J_wf({\mathcal {L}}-y_0)+J_yf(y_0), \end{aligned}$$

and for the reduced model:

77 $$\begin{aligned} J'_a= & {} J'_bf({\mathcal {L}})(2p_\mathrm{S}-1)+f(x_0)(2p_\mathrm{S}-1), \end{aligned}$$

78 $$\begin{aligned} J'_b= & {} J'_{d}f(l_0)2p_\mathrm{D}+J'_af({\mathcal {L}})(2p_\mathrm{D}-1)\nonumber \\&+\,f({\mathcal {L}}-x_0)(2p_\mathrm{D}-1), \end{aligned}$$

79 $$\begin{aligned} J'_c= & {} J_af({\mathcal {L}})2p'_\mathrm{T}+J'_df(l_0)(2p'_\mathrm{T}-1)+f({\mathcal {L}})2p'_\mathrm{T},\end{aligned}$$

80 $$\begin{aligned} J'_{d}= & {} J'_{c}f(l_0)(-p'_\mathrm{GJ})+J'_{t}f(l_0)p'_\mathrm{GJ},\end{aligned}$$

81 $$\begin{aligned} J'_{s}= & {} J'_{c}f(l_0)p'_\mathrm{GJ}+J'_{t}f(l_0)(-p'_\mathrm{GJ}),\end{aligned}$$

82 $$\begin{aligned} J'_t= & {} J_yf({\mathcal {L}})2p'_\mathrm{T}+J'_sf(l_0)(2p'_\mathrm{T}-1),\end{aligned}$$

83 $$\begin{aligned} J'_w= & {} J'_{s}f(l_0)2p_\mathrm{D}+J_yf({\mathcal {L}})(2p_\mathrm{D}-1),\end{aligned}$$

84 $$\begin{aligned} J'_y= & {} J'_wf({\mathcal {L}})(2p_\mathrm{S}-1),\end{aligned}$$

85 $$\begin{aligned} J'_{y_0}= & {} J'_wf({\mathcal {L}}-y_0)+J'_yf(y_0). \end{aligned}$$

Indices *i* and *j* in the equations for the full model change from 1 to $n_\mathrm{GJ}$, and $p_\mathrm{T}$ is a branch factor of any tuft dendrite defined as in (16).

It is possible to prove that Eq. (67) holds and the systems in Fig. 12a, b are equivalent when, in addition to constraints (63)–(66), $p'_\mathrm{T}=n_\mathrm{GJ} p_\mathrm{T}$, $R'_\mathrm{GJ}=R_\mathrm{GJ}/n_\mathrm{GJ}$ and $z'=n_\mathrm{GJ}z$, giving

86 $$\begin{aligned} p_\mathrm{GJ}'=p_\mathrm{GJ}=\frac{1}{2+2zR_\mathrm{GJ}}, \end{aligned}$$

which constitutes part of the definition of a trip coefficient for the GJ node (see Fig. 2c). A detailed proof of model reduction is given in “Appendix 3”.

Our method for constructing the compact Green’s functions can then be simply applied to the reduced model shown in Fig. 12b. A detailed derivation of the solutions for the model with a stimulation applied at $y_0$ in Cell 1 and the output points $x_0$ placed in both cells is given in “Appendix 4”. Assuming that $y_0$ is placed at the soma of Cell 1, the Green’s functions at each soma have the following forms

87 $$\begin{aligned} G_{2}(0,0)= & {} \frac{\theta \eta ^2p_\mathrm{D}p_\mathrm{S}^2p_\mathrm{GJ}f({\mathcal {L}}+2l_0)}{D\gamma (1+2\mu )}, \end{aligned}$$

88 $$\begin{aligned} G_{1}(0,0)= & {} \frac{p_\mathrm{S}}{2D\gamma }(1+\delta f({\mathcal {L}}))\eta -G_{2}(0,0), \end{aligned}$$

where $$\zeta =f({\mathcal {L}})(2p_\mathrm{S}-1), \delta =f({\mathcal {L}})(2p_\mathrm{D}-1)$$,

$$\theta =2n_\mathrm{GJ}p_\mathrm{T}f({\mathcal {L}}), \eta =2/(1-\zeta \delta )$$ and $$\mu =(\zeta \theta \eta p_\mathrm{D}+2n_\mathrm{GJ}p_\mathrm{T}-1)p_\mathrm{GJ}f(2l_0)$$.

For investigating the effect of gap junctions from the tufted regions of the cells on the model’s behaviour, we define a coupling ratio (CR) as

89 $$\begin{aligned} \text {CR}=\frac{\max _t\text {InvLT}\{G_{2}(0,0;\omega )\}(t)}{\max _t\text {InvLT}\{G_{1}(0,0;\omega )\}(t)}. \end{aligned}$$

**Fig. 13:**
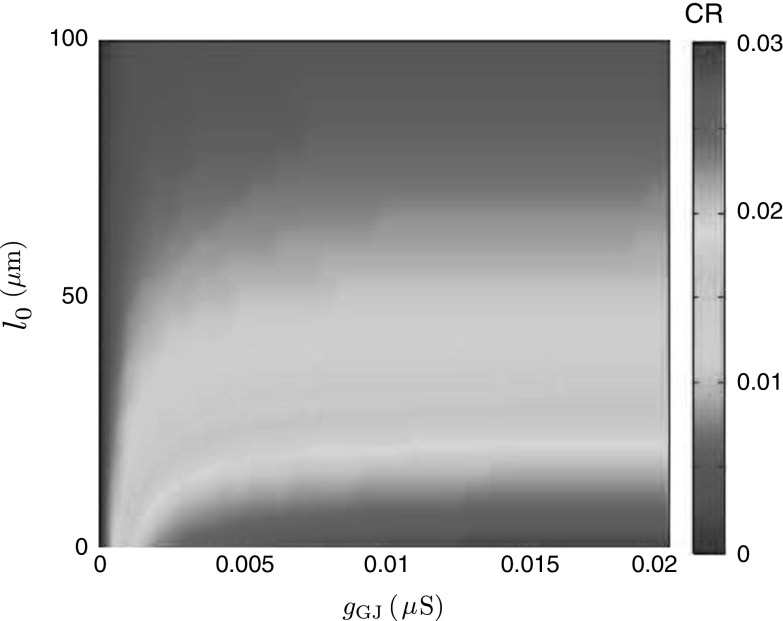
Coupling ratio as a function of gap-junctional conductances and distances from the branch point with the primary dendrite. Both cells are identical and passive. Dendritic parameters: $a=0.4\,\mu \mathrm{m}$, $C=1\,\mu \mathrm{F}\cdot \hbox {cm}^{-2}$, $R=2000\,\Omega \cdot \hbox {cm}^2$, $R_{a}=150\,\Omega \cdot \hbox {cm}$. Somatic parameters: $a_\mathrm{S}=25\,\mu \mathrm{m}$, $C_\mathrm{soma}=1\,\mu \mathrm{F}\cdot \hbox {cm}^{-2}$, $R_\mathrm{soma}=2000\,\Omega \cdot \hbox {cm}^2$. The length of the primary dendrite is ${\mathcal {L}}=350\,\mu \mathrm{m}$

Using Eqs. (87) and (88), we compute and plot in Fig. 13 a map of CR for various values of conductance $g_\mathrm{GJ}$ and location $l_{0}$ of the gap junctions in the tuft. This map can be compared with the CR map obtained earlier in [12] for two mitral cells coupled by distal gap junctions. Note that the map in [12] is obtained by brute-force numerical simulations of a computational model with a similar, but not identical, structure to our two-cell model.

**Fig. 14:**
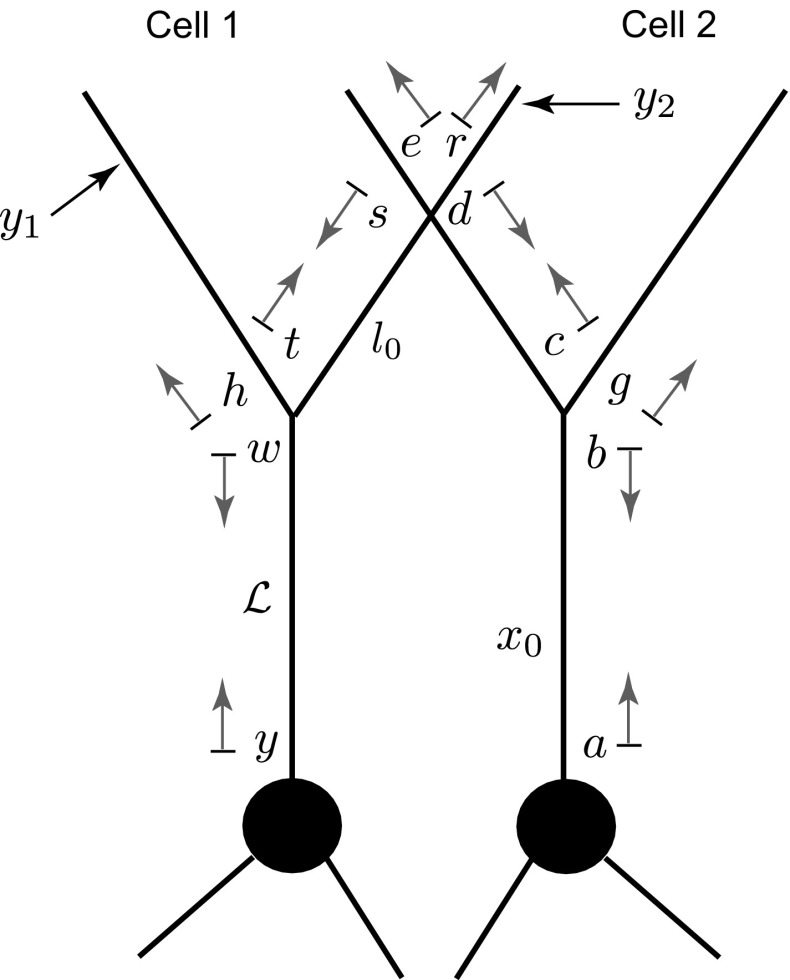
A reduced model with inputs applied to the tuft

Using our method of local point matching, we can also prove that there exists an equivalent reduced model for the full tufted model with external inputs applied to the tufts (instead of the primary dendrites). We consider that any tuft dendrite *k* can receive a Dirac delta pulse at the location $y_k$ away from the branch point with the primary dendrite. This tuft dendrite can be either with or without a gap junction. In the equivalent reduced model shown in Fig. 14, we consider two possible inputs corresponding to the location of $y_k$, namely the input $y_1$ applied to the branch without a gap junction and $y_2$ applied to the branch with a gap junction.

It is possible to show (see “Appendix 5” for details) that the Green’s function of the full tufted model for a given input $y_k$ can be found knowing the Green’s function for the equivalent reduced model as

90 $$\begin{aligned} G(x_0,y_k)=\frac{1}{n_\mathrm{T}-n_\mathrm{GJ}}G'(x_0,y_1), \end{aligned}$$

for the input $y_k$ applied to the branch without a gap junction, and

91 $$\begin{aligned} G(x_0,y_k)=\frac{1}{n_\mathrm{GJ}}G'(x_0,y_2), \end{aligned}$$

for the input $y_k$ applied to the branch with a gap junction. Here, the reduced model is constructed in such a way that the stimuli in the full and reduced models are located at the same distance away from the primary dendrites, i.e. $y_{1}=y_{k}$ and $y_{2}=y_{k}$. The point $x_0$ ($0 \le x_0 \le {\mathcal {L}}$) is located on the primary dendrite of either of two cells. The reduced model shown in Fig. 14 can be constructed from the full tufted model using a number of constraints specified in (164)–(168) and (174). The Green’s functions $G'$ for each cell can then be found by our method of local point matching and used in (90) and (91) for finding the Green’s functions for the full tufted model.

In the case of multiple inputs applied to the tuft dendrites, the Green’s function for each cell can be found by summing individual Green’s functions for each input. Assuming that all tuft dendrites of both cells receive identical inputs located at a distance *y* away from the primary dendrite of each cell, we obtain in this special case

92 $$\begin{aligned} G(x_0,y)=G'(x_0,y). \end{aligned}$$

## Discussion

In this paper we have presented a novel method for calculating the Green’s functions for arbitrary neuronal networks with a passive or resonant cell membrane coupled by dendro-dendritic gap junctions. This method provides an alternative and complementary approach to the generalised sum-over-trip method [19]. Importantly, our new approach avoids the construction of an infinite number of trips and, being based on the construction of a linear system of algebraic equations, provides exact expressions for the network response function in the Laplace (frequency) domain without any issues of computational convergence. We have applied this new method of local point matching to a simple single cell model and two-cell neuronal networks (simplified and with tuft dendrites). Its application to the tufted network has also allowed us to reduce it to an equivalent network, but with a much simpler morphological structure. We have also illustrated that knowledge of the exact compact expressions for the Green’s function can provide important insights into the role of individual variations in cell parameters on the model’s dynamics.

There are a number of natural extensions of the work in this paper. One is an application to more realistic network geometries with more than just two cells, given that a computational implementation of the method of local point matching can provide a fast realisation of the Green’s function for the whole network. Having a complex network of multiple cells with a graph structure consisting of *N* dendritic segments, we need to construct and solve a linear system of 2*N* equations only once to find all unknown $J_{v_{k}}$ and $J_{w_{k}}$ functions. We can then simply construct the functions $J_{y}$ for each dendritic segment to obtain $G_{ij}(X,Y;\omega )$. Note that the point *X* can be placed on each dendritic segment before constructing a system of linear equations for $J_{v_{k}}$ and $J_{w_{k}}$. Switching off all *X* points except one on branch *i* in the solution for $J_{y}$ allows one to find the Green’s function for the entire network straight away. The numerical inverse Laplace transform to obtain ${\widehat{G}}_{ij}(X,Y;t)$ is the only procedure in which a computational approximation appears. As has been previously pointed in Sect. 4.2, knowledge of a map from the preferred frequencies to the system’s parameters for a reconstructed neuronal network combined with subthreshold electrophysiological data might provide some estimates for important network’s parameters and additional work is required in this direction. Another possible extension is to incorporate active properties in dendrites and somas of cells in a network and analyse the propagation of dendritic action potentials as well as somatic spiking dynamics. The spike-diffuse-spike (SDS)-type model [5, 17] can be utilised for that, as although the voltage-gated channels in the SDS framework are modelled by piecewise linear instead of nonlinear dynamics, it has been shown that the speed of wave propagation in the SDS model is in excellent agreement with a more biophysically realistic nonlinear model [20]. Both these extensions will be reported on elsewhere.

## Appendix 1: Derivation of a method of local point matching

Introducing the function

93 $$\begin{aligned} J_{ij}(x,y;\omega )=2D_j\gamma _j(\omega ){G}_{ij}(x,y;\omega ), \end{aligned}$$

we can write from Eq. (15) that

94 $$\begin{aligned} J_{ij}(x,y;\omega )=\sum _\mathrm{trips}A_\mathrm{trip}(\omega )f(L_\mathrm{trip}(x,y;\omega )). \end{aligned}$$

We assume that two points $v_j$ and $w_j$ are placed on segment *j* infinitesimally close to each end of it such that the point *y* which is not at a node (i.e. $0<y<{\mathcal {L}}_j$) is between $v_j$ and $w_j$. Then $J_{ij}(x,y;\omega )$ can be found as a combination of two classes of trips:

95 $$\begin{aligned} J_{ij}(x,y)&=\sum _{\mathrm{trips}}A_{v_j}f(L_\mathrm{trip}(x,y))\nonumber \\&\qquad +\sum _{\mathrm{trips}}A_{w_j}f(L_\mathrm{trip}(x,y)) \end{aligned}$$

96 $$\begin{aligned}&\quad =f(v_j-y)\sum _\mathrm{trips}A_{v_j}f(L_\mathrm{trip}(x,v_j^{\rightarrow y}))\nonumber \\&\quad \quad +f(w_j-y)\sum _\mathrm{trips}A_{w_j}f(L_\mathrm{trip}(x,w_j^{\rightarrow y})). \end{aligned}$$

Note that $\omega$ is omitted for compactness from this point. The trips in (95) are divided into two groups: the trips that are passing through $v_j$ just before reaching *y* and the trips that are passing through $w_j$ just before reaching *y* (see Fig. 3). $L_\mathrm{trip}(x,v_j^{\rightarrow y})$ introduced in (96) defines a length of a trip which moves in the direction of *y* and ends at $v_j$ before reaching *y*. Similarly, $L_\mathrm{trip}(x,w_j^{\rightarrow y})$ defines a length of a trip which moves in the direction of *y* and ends at $w_j$ before reaching *y* (shown in red in Fig. 3). $A_{v_j}$ and $A_{w_j}$ are the trip coefficients corresponding to the trips to $v_j$ and $w_j$ and obtained using the rules of the sum-over-trips method.

As $v_j$ is placed close to one end of the segment, we have $L_\mathrm{trip}(x,v_j)=L_\mathrm{trip}(x,v_j^{\rightarrow y})$, and therefore, we introduce

97 $$\begin{aligned} J_{ij}(x,v_j)=\sum _\mathrm{trips}A_{v_j}f(L_\mathrm{trip}(x,v_j^{\rightarrow y})). \end{aligned}$$

Similarly, as $w_j$ is located close to the other end of the segment, we introduce

98 $$\begin{aligned} J_{ij}(x,w_j)=\sum _\mathrm{trips}A_{w_j}f(L_\mathrm{trip}(x,w_j^{\rightarrow y})). \end{aligned}$$

Then simplifying the notations as $J_{ij}(x,y)=J_y$,

$J_{ij}(x,v_j)=J_{v_j}$ and $J_{ij}(x,w_j)=J_{w_j}$ we can rewrite Eq. (96) in the form

99 $$\begin{aligned} J_y=f(v_j-y)J_{v_j}+f(w_j-y)J_{w_j}. \end{aligned}$$

As both points $v_j$ and $w_j$ are placed infinitesimally close to individual ends of segment *j* of length ${\mathcal {L}}_j$, we consider that $v_j=0$ and $w_j={\mathcal {L}}_j$, and therefore, Eq. (99) can be rewritten as

100 $$\begin{aligned} J_y=f(y)J_{v_j}+f({\mathcal {L}}_j-y)J_{w_j}. \end{aligned}$$

If the point *y* is located on a semi-infinite branch and $w_j$ is placed on the side towards infinity, then $|w_j-y|\rightarrow \infty$ giving $f(w_j-y)J_{w_j}=0$.

Following similar steps, if placing two points $v_k$ and $w_k$ on each segment *k* infinitesimally close to each end, we can define functions $J_{v_k}$ and $J_{w_k}$ which can be written in terms of functions $J_{v_n}$ and $J_{w_n}$ associated with points $v_{n}$ and $w_{n}$ from all branches connected to a single node. For example, given a node with *K* segments and *K* pairs of points $(v_k,w_k)$ (see Fig. 4), the function $J_{v_k}$ can be found as

101 $$\begin{aligned} J_{v_k}= & {} \sum _{n=1}^{K}\sum _\mathrm{trips}A_{w_n}f(L_\mathrm{trip}(x,w_n))a_{nk}f({\mathcal {L}}_n)\nonumber \\= & {} \sum _{n=1}^{K}a_{nk}f({\mathcal {L}}_n)\sum _\mathrm{trips}A_{w_n}f(L_\mathrm{trip}(x,w_n))\nonumber \\= & {} \sum _{n=1}^{K}a_{nk}f({\mathcal {L}}_n) J_{w_n}, \qquad k=1,\dots ,~K, \end{aligned}$$

where $a_{nk}$ is a coefficient for a trip passing from segment *n* to segment *k*, $a_{kk}$ is a coefficient for a trip reflecting at one of the ends of segment *k* and ${\mathcal {L}}_n$ is the scaled length of branch *n*. Equation (101) can be constructed for any node branches of which do not include point *x*. If *x* is located on branch *i* connected to a node in consideration ($0<x<{\mathcal {L}}_i$), an additional term representing a trip from *x* to $v_k$ needs to be added:

102 $$\begin{aligned} J_{v_k}=\sum _{n=1}^{K}a_{nk}f({\mathcal {L}}_n) J_{w_n}+a_{ik}f(x). \end{aligned}$$

For a given network, the number of functions $$J_{v}$$ and $$J_{w}$$ is equal to the degree sum of the corresponding graph and a system of linear equations for unknown $$J_{v}$$ and $$J_{w}$$ can be written using Eqs. (101) and (102). It is possible to show that this system of equations is linearly independent and therefore has a unique solution. Solving it, we can find $$J_{v_j}$$ and $$J_{w_j}$$ and obtain $$J_{y}=J_{ij}(x,y)$$ from Eq. (100). The Green’s function $$ G_{ij}(x,y)$$ can then be calculated from Eq. (93) as

103 $$\begin{aligned} G_{ij}(x,y)=\frac{J_{y}}{2D_{j}\gamma _{j}}. \end{aligned}$$

If both *x* and *y* are located on the same branch, equation for $J_{y}$ takes the following form (instead of Eq. 100):

104 $$\begin{aligned} J_y=f(y)J_{v_j}+f({\mathcal {L}}_j-y)J_{w_j}+f(x-y). \end{aligned}$$

## Appendix 2: Solving a simplified two-cell network model

Equations (37) and (39) give

105 $$\begin{aligned} J_w+J_b=0. \end{aligned}$$

Then using Eqs. (36), (38) and (105) we can get

106 $$\begin{aligned} J_y+J_a=f(x_2)(2p_\mathrm{S}-1). \end{aligned}$$

Using Eqs. (38), (39) and (106), we can obtain that

107 $$\begin{aligned} J_w= & {} \frac{p_\mathrm{GJ} f({\mathcal {L}})}{q}[f(x_2)(2p_\mathrm{S}-1)+1/f(x_2)]\nonumber \\= & {} \frac{p_\mathrm{GJ} f({\mathcal {L}})}{q}{\widetilde{F}}(x_2,0), \end{aligned}$$

where function ${\widetilde{F}}$ is defined in (46).

Placing the stimulus to the point $y_{1}$ (see Fig. 8), we have

108 $$\begin{aligned} J_{y_1}=J_z f(y_1-{\mathcal {L}}_\mathrm{GJ}) \end{aligned}$$

with

109 $$\begin{aligned} J_z=J_y f({\mathcal {L}}_\mathrm{GJ})(1-p_\mathrm{GJ})+J_a f({\mathcal {L}}_\mathrm{GJ}) p_\mathrm{GJ}+f({\mathcal {L}}_\mathrm{GJ}-x_2)p_\mathrm{GJ}, \end{aligned}$$

and the solution $G_{2}(x_2,y_1)$ can be found as

110 $$\begin{aligned} G_{2}(x_2,y_1)=\frac{J_{y_{1}}}{2D\gamma }=\frac{1}{2D\gamma }\frac{p_\mathrm{GJ}+p_\mathrm{GJ}\alpha }{q}{\widetilde{F}}(x_2,y_1). \end{aligned}$$

Similarly for $y_{2}$, we can show that

111 $$\begin{aligned} G_{2}(x_2,y_2)=\frac{J_{y_{2}}}{2D\gamma }=\frac{1}{2D\gamma }\frac{1-p_\mathrm{GJ}+p_\mathrm{GJ}\alpha }{q}{\widetilde{F}}(x_2,y_2). \end{aligned}$$

Solutions $G_{1}(x_1,y_2)$ and $G_{1}(x_1,y_1)$ can be easily obtained using the symmetry of the system. Note that for the case of two symmetrical inputs located after the gap junction (i.e. $y_1=y_2=y$), we have

112 $$\begin{aligned} G_{1}(x_1,y_{1})+G_{1}(x_1,y_{2})= & {} \frac{{\widetilde{F}}(x_1,y_{1}=y)}{2D\gamma }, \end{aligned}$$

113 $$\begin{aligned} G_2(x_2,y_{1})+G_2(x_2,y_{2})= & {} \frac{{\widetilde{F}}(x_2,y_{2}=y)}{2D\gamma }. \end{aligned}$$

When the stimulus is applied to the location $y_3$, we can get that

114 $$\begin{aligned} J_{y_3}=J_y f(y_3)+J_w f({\mathcal {L}}_\mathrm{GJ}-y_3), \end{aligned}$$

and therefore obtain

115 $$\begin{aligned} G_{2}(x_2,y_3)= & {} \frac{J_{y_3}}{2D\gamma }\nonumber \\= & {} \frac{1}{2D\gamma }\frac{p_\mathrm{GJ}f(2{\mathcal {L}}_\mathrm{GJ})}{q}{\widetilde{F}}(x_2,0){\widetilde{F}}(y_3,0). \end{aligned}$$

Considering the stimulus at the point $y_4$, we have

116 $$\begin{aligned} J_{y_4}=J_{a}f(y_4)+J_b f({\mathcal {L}}_\mathrm{GJ}-y_4)+ f(y_4-x_2). \end{aligned}$$

To find $G_2(x_2,y_4)$, we notice that if two symmetrical inputs ($y_3=y_4=y$) are applied to the system, then using Eqs. (114), (116), (105) and (106), we get

117 $$\begin{aligned}&G_{2}(x_2,y_3=y)+G_{2}(x_2,y_4=y)\nonumber \\&\quad =\frac{1}{2D\gamma }(J_{y_3=y}+J_{y_4=y})=\frac{1}{2D\gamma }(f(x_2+y)(2p_\mathrm{S}-1)\nonumber \\&\qquad +f(x_2-y)). \end{aligned}$$

From (117) and (115), we can then find

118 $$\begin{aligned}&G_{2}(x_2,y_4)=\frac{1}{2D\gamma }\bigg [f(x_2+y_4)(2p_\mathrm{S}-1)+f(x_2-y_4)\nonumber \\&\qquad \qquad \quad \qquad -\frac{p_\mathrm{GJ}f(2{\mathcal {L}}_\mathrm{GJ})}{q}{\widetilde{F}}(x_2,0){\widetilde{F}}(y_4,0)\bigg ]\nonumber \\&={\left\{ \begin{array}{ll} \frac{1}{2D\gamma }\big [{\widetilde{F}}(x_2,y_4)\\ -\frac{p_\mathrm{GJ}f(2{\mathcal {L}}_\mathrm{GJ})}{q}{\widetilde{F}}(x_2,0){\widetilde{F}}(y_4,0)\big ],\quad \text {if }x_2<y_4,\\ \frac{1}{2D\gamma }\big [{\widetilde{F}}(y_4,x_2)\\ -\frac{p_\mathrm{GJ}f(2{\mathcal {L}}_\mathrm{GJ})}{q}{\widetilde{F}}(x_2,0){\widetilde{F}}(y_4,0)\big ],\quad \text {if } x_2>y_4. \end{array}\right. }\nonumber \\ \end{aligned}$$

Solutions $G_1(x_1,y_3)$ and $G_1(x_1,y_4)$ can be easily found using the symmetry.

## Appendix 3: Model reduction in a two-cell tufted network

In the case of the model with the input/output points placed in the primary dendrite, it is straightforward to demonstrate using our algorithm that $n_\mathrm{T}-n_\mathrm{GJ}$ branches having no gap junctions can be easily merged into a single branch. From Eqs. (75), (76), (84) and (85) we obtain that

119 $$\begin{aligned} J_{y_0}= & {} J_wf({\mathcal {L}}) 2p_\mathrm{S}, \end{aligned}$$

120 $$\begin{aligned} J'_{y_0}= & {} J'_wf({\mathcal {L}}) 2p_\mathrm{S}. \end{aligned}$$

Then due to Eq. (67), we get

121 $$\begin{aligned} J'_w=J_w, \end{aligned}$$

and then

122 $$\begin{aligned} J'_y=J_y. \end{aligned}$$

Comparing Eqs. (74) and (83) with the equality (121), we obtain

123 $$\begin{aligned} J'_s=\sum _{i} J_{s_i}, \qquad i=1,\dots ,~n_\mathrm{GJ}. \end{aligned}$$

Equations (71), (72), (80) and (81) give

124 $$\begin{aligned} J_{d_i}+J_{s_i}= & {} 0, \end{aligned}$$

125 $$\begin{aligned} J'_{d}+J'_{s}= & {} 0, \end{aligned}$$

and with relationship (123) we have

126 $$\begin{aligned} J'_d=\sum J_{d_i}. \end{aligned}$$

Using Eqs. (68), (69), (77) and (78), we can obtain

127 $$\begin{aligned}&\big [1-f(2{\mathcal {L}})(2p_\mathrm{S}-1)(2p_\mathrm{D}-1)\big ]J_b\nonumber \\&\quad =\sum _{i} J_{d_i}f(l_0)2p_\mathrm{D}+f({\mathcal {L}})(2p_\mathrm{D}-1)2p_\mathrm{S}, \end{aligned}$$

128 $$\begin{aligned}&\big [1-f(2{\mathcal {L}})(2p_\mathrm{S}-1)(2p_\mathrm{D}-1)\big ]J'_b\nonumber \\&\quad =J'_df(l_0)2p_\mathrm{D}+f({\mathcal {L}})(2p_\mathrm{D}-1)2p_\mathrm{S}, \end{aligned}$$

and together with (126) we have

129 $$\begin{aligned} J'_b=J_b, \end{aligned}$$

and then

130 $$\begin{aligned} J'_a=J_a. \end{aligned}$$

Using the relationships (123) and (126) we can rewrite Eqs. (70) and (73) as

131 $$\begin{aligned} J_{c_i}= & {} J_af({\mathcal {L}})2p_\mathrm{T}+J'_d f(l_0)2p_\mathrm{T}-J_{d_i}f(l_0)\nonumber \\&+f({\mathcal {L}})2p_\mathrm{T}, \end{aligned}$$

132 $$\begin{aligned} J_{t_i}= & {} J_yf({\mathcal {L}})2p_\mathrm{T}+J'_s f(l_0)2p_\mathrm{T}- J_{s_i}f(l_0), \end{aligned}$$

and after summing all functions $$ J_{c_i}$$ and $$J_{t_i}$$ over $$i=1,\dots ,~n_\mathrm{GJ}$$, we get

133 $$\begin{aligned} \sum _i J_{c_i}= & {} J_af({\mathcal {L}})2\sum _i p_\mathrm{T}+J'_d f(l_0)\bigg (2\sum _i p_\mathrm{T}-1\bigg )\nonumber \\&+f({\mathcal {L}})2\sum p_\mathrm{T}, \end{aligned}$$

134 $$\begin{aligned} \sum _i J_{t_i}= & {} J_yf({\mathcal {L}})2\sum _i p_\mathrm{T}+J'_s f(l_0)\bigg (2\sum _i p_\mathrm{T}-1\bigg ).\nonumber \\ \end{aligned}$$

Right-hand sides of these equations have the same forms as Eqs. (79) and (82) for the functions $J'_{c}$ and $J'_{t}$ under the constraint

135 $$\begin{aligned} p'_\mathrm{T}=\sum _i p_\mathrm{T}. \end{aligned}$$

Therefore, we have

136 $$\begin{aligned} J'_c= & {} \sum _i J_{c_i}, \end{aligned}$$

137 $$\begin{aligned} J'_t= & {} \sum _i J_{t_i}. \end{aligned}$$

We can also sum Eqs. (71) and (72) over index $i=1,\dots ,~n_\mathrm{GJ}$ to obtain

138 $$\begin{aligned} \sum _i J_{d_i}= & {} \sum _i J_{c_i}f(l_0)(-p_\mathrm{GJ})+\sum _i J_{t_i}f(l_0)p_\mathrm{GJ}, \end{aligned}$$

139 $$\begin{aligned} \sum _i J_{s_i}= & {} \sum _i J_{c_i}f(l_0)p_\mathrm{GJ}+\sum _i J_{t_i}f(l_0)(-p_\mathrm{GJ}), \end{aligned}$$

and compare them with Eqs. (80) and (81). The reduced model will then be equivalent to the full tufted model if, in addition to the previous constraints, the following relationship holds

140 $$\begin{aligned} p'_\mathrm{GJ}=p_\mathrm{GJ}, \end{aligned}$$

leading to $R'_\mathrm{GJ}=R_\mathrm{GJ}/n_\mathrm{GJ}$ and $z'=n_\mathrm{GJ}z$.

## Appendix 4: Closed form solution to the tufted model

Solving Eqs. (77)–(84) for the reduced model and applying the constraints we get

141 $$\begin{aligned} J'_a= & {} \eta p_\mathrm{S}-1-\frac{\zeta \theta \eta ^2p_\mathrm{GJ}p_\mathrm{S}p_\mathrm{D}f(2l_0)}{1+2\mu }, \end{aligned}$$

142 $$\begin{aligned} J'_b= & {} \delta \eta p_\mathrm{S}-\frac{\theta \eta ^2p_\mathrm{GJ}p_\mathrm{S}p_\mathrm{D}f(2l_0)}{1+2\mu },\end{aligned}$$

143 $$\begin{aligned} J'_c= & {} \theta \eta p_\mathrm{S}\frac{1+\mu }{1+2\mu },\end{aligned}$$

144 $$\begin{aligned} J'_d= & {} \frac{-\theta \eta p_\mathrm{GJ}p_\mathrm{S}f(l_0)}{1+2\mu },\end{aligned}$$

145 $$\begin{aligned} J'_s= & {} \frac{\theta \eta p_\mathrm{GJ}p_\mathrm{S}f(l_0)}{1+2\mu },\end{aligned}$$

146 $$\begin{aligned} J'_t= & {} \theta \eta p_\mathrm{S}\frac{\mu }{1+2\mu },\end{aligned}$$

147 $$\begin{aligned} J'_w= & {} \frac{\theta \eta ^2p_\mathrm{GJ}p_\mathrm{S}p_\mathrm{D}f(2l_0)}{1+2\mu },\end{aligned}$$

148 $$\begin{aligned} J'_y= & {} \frac{\zeta \theta \eta ^2p_\mathrm{GJ}p_\mathrm{S}p_\mathrm{D}f(2l_0)}{1+2\mu }. \end{aligned}$$

Placing $y_{0}$ and $x_{0}$ on the primary dendrites of Cell 1 and Cell 2, respectively, we can write that

149 $$\begin{aligned} \begin{aligned} J_{y_0}=J'_{y_0}&=J'_wf({\mathcal {L}})+J'_yf(0)\\&=\frac{2\theta \eta ^2p_\mathrm{D}p_\mathrm{S}^2p_\mathrm{GJ}f({\mathcal {L}}+2l_0)}{1+2\mu }. \end{aligned} \end{aligned}$$

As all dendrites in the tuft are identical, Eq. (135) is simply $p'_\mathrm{T}=n_\mathrm{GJ} p_\mathrm{T}$. Using this relationship in (149) and having $x_{0}=y_{0}=0$, we obtain the somatic Green’s function for Cell 2 (Eq. 87).

As the cells are identical and the system is symmetrical, the Green’s function for Cell 1 (where the input is placed) is equal to the Green’s function for Cell 2 if both $x_{0}$ and $y_{0}$ are placed on Cell 2. For this case $J_{y_0}$ takes the form

150 $$\begin{aligned} J_{y_0}=J'_{y_0}=J'_b f({\mathcal {L}})+J'_a f(0)+f(0). \end{aligned}$$

Then

151 $$\begin{aligned}&G_2(0,0)+G_1(0,0)=\frac{1}{2D\gamma }[J'_wf({\mathcal {L}})+J'_yf(0)\nonumber \\&\quad +\,J'_b f({\mathcal {L}})+J'_a f(0)+f(0)]=\frac{ p_\mathrm{S}}{2D\gamma }(1+\delta f({\mathcal {L}}))\eta ,\nonumber \\ \end{aligned}$$

which gives Eq. (88).

Note that by setting $p_\mathrm{D}=1/2$ which gives $\delta =0$, $p'_\mathrm{T}=1/2$, $\theta =f({\mathcal {L}})$, we can recover a case of two simplified identical cells (without the tuft). Equation (87) gives

152 $$\begin{aligned} G_{2}(0,0)=\frac{1}{D\gamma }\frac{2p_\mathrm{S}^2p_\mathrm{GJ}f(2{\mathcal {L}}+2l_0)}{1+2(2p_\mathrm{S}-1)p_\mathrm{GJ}f(2{\mathcal {L}}+2l_0)}, \end{aligned}$$

and this is exactly Eq. (42) with $x_2=0$, $y_3=0$ and ${\mathcal {L}}_\mathrm{GJ}={\mathcal {L}}+l_0$.

## Appendix 5: Inputs applied to the tuft

Here we assume that a single input is placed on segment *k* of the tuft of Cell 1 at the distance $y_{k}$ away from the branch point with the primary dendrite (see Fig. 14 for a reduced version of the model). Two cases are possible: (i) this segment *k* is uncoupled from Cell 2 and (ii) this segment *k* is coupled by a gap junction with Cell 2.

As the tufted branches are identical, using the constraints (123), (126), (136) and (137), we have

153 $$\begin{aligned} J'_{c}= & {} n_\mathrm{GJ} J_{c_i}, \end{aligned}$$

154 $$\begin{aligned} J'_{d}= & {} n_\mathrm{GJ} J_{d_i},\end{aligned}$$

155 $$\begin{aligned} J'_{s}= & {} n_\mathrm{GJ} J_{s_i},\end{aligned}$$

156 $$\begin{aligned} J'_{t}= & {} n_\mathrm{GJ} J_{t_i},\end{aligned}$$

157 $$\begin{aligned} J'_{e}= & {} (n_\mathrm{T}-n_\mathrm{GJ}) J_{e_i},\end{aligned}$$

158 $$\begin{aligned} J'_{g}= & {} (n_\mathrm{T}-n_\mathrm{GJ}) J_{g_i},\end{aligned}$$

159 $$\begin{aligned} J'_{h}= & {} (n_\mathrm{T}-n_\mathrm{GJ}) J_{h_i},\end{aligned}$$

160 $$\begin{aligned} J'_{r}= & {} (n_\mathrm{T}-n_\mathrm{GJ}) J_{r_i} \end{aligned}$$

Skipping the subscript *i* used in the full model, we get for case (i) when, for example, the input is located on a branch with point *h* as in Fig. 14:

161 $$\begin{aligned} J_{y_k}= & {} J_{h}f(y_k),\end{aligned}$$

162 $$\begin{aligned} J'_{y_1}= & {} J'_{h}f(y_1). \end{aligned}$$

This gives

163 $$\begin{aligned} J_{y_k}=\frac{J'_{y_1}}{n_\mathrm{T}-n_\mathrm{GJ}}. \end{aligned}$$

Using relations (66) and

164 $$\begin{aligned} p'_{\mathrm{T}\_\mathrm{GJ}}=\sum _i^{n_\mathrm{GJ}} p_{\mathrm{T}\_\mathrm{GJ}}, \end{aligned}$$

which is just Eq. (135) for the tuft dendrites with gap junctions, we can obtain that

165 $$\begin{aligned} p'_{\mathrm{T}\_\mathrm{noGJ}}=\sum _i^{n_\mathrm{T}-n_\mathrm{GJ}} p_{\mathrm{T}\_\mathrm{noGJ}}=(n_\mathrm{T}-n_\mathrm{GJ})p_{\mathrm{T}\_\mathrm{noGJ}}, \end{aligned}$$

where $p_{\mathrm{T}\_\mathrm{noGJ}}$ and $p'_{\mathrm{T}\_\mathrm{noGJ}}$ are branch factors of any tuft dendrite without gap junctions in the full and reduced models, respectively. From constraints (66), (164) and (165), we obtain the additional constraints for the models’ equivalence:

166 $$\begin{aligned}&z'_\mathrm{D}=z_\mathrm{D}, \end{aligned}$$

167 $$\begin{aligned}&z'_{\mathrm{T}\_\mathrm{GJ}}=n_\mathrm{GJ} z_{\mathrm{T}\_\mathrm{GJ}}, \end{aligned}$$

168 $$\begin{aligned}&z'_{\mathrm{T}\_\mathrm{noGJ}}=(n_\mathrm{T}-n_\mathrm{GJ}) z_{\mathrm{T}\_\mathrm{noGJ}}, \end{aligned}$$

where $z=\gamma (\omega )/r_a$ defined as in (16) are given for the primary dendrite ($\mathrm{D}$), any tuft dendrite with a gap junction (T_GJ) and any tuft dendrite without a gap junction (T_noGJ). These constraints (166)–(168) can be achieved by controlling diameters of the corresponding branches (i.e. by varying $r_a$ parameter) and keeping identical electrical properties of cell membrane (i.e. $\gamma (\omega )$) in both the full and reduced models. Then Eq. (163) gives

169 $$\begin{aligned} G(x_0,y_k)=\frac{1}{n_\mathrm{T}-n_\mathrm{GJ}}G'(x_0,y_1). \end{aligned}$$

Considering case (ii) when the input is placed on the tuft dendrite with a gap junction (stimulus $y_2$ in the reduced model in Fig. 14), we can get that

170 $$\begin{aligned} J_{y_k}= & {} J_tf(y_k)+J_sf(l_0-y_k),\quad \text {if}~y_k<l_0, \end{aligned}$$

171 $$\begin{aligned} J'_{y_2}= & {} J'_tf(y_2)+J'_sf(l_0-y_2), \quad \text {if}~y_2<l_0, \end{aligned}$$

and

172 $$\begin{aligned} J_{y_k}= & {} J_rf(y_k-l_0)=[J_cf(l_0)p_\mathrm{GJ}\nonumber \\&+\,J_tf(l_0)(1-p_\mathrm{GJ})]f(y_k-l_0)\nonumber \\= & {} (J_cp_\mathrm{GJ}+J_t(1-p_\mathrm{GJ}))f(y_k),\quad \text {if}~y_k>l_0, \end{aligned}$$

173 $$\begin{aligned} J'_{y_2}= & {} J'_rf(y_0-l_0)=[J'_cf(l_0)p'_\mathrm{GJ}\nonumber \\&+\,J'_tf(l_0)(1-p'_\mathrm{GJ})]f(y_0-l_0)\nonumber \\= & {} (J'_cp'_\mathrm{GJ}+J'_t(1-p'_\mathrm{GJ}))f(y_0),\quad \text {if}~y_2>l_0. \end{aligned}$$

Then adding a constraint [notably the same as Eq. (140) in the previous model]

174 $$\begin{aligned} p'_\mathrm{GJ}=p_\mathrm{GJ}, \end{aligned}$$

we have

175 $$\begin{aligned} J_{y_k}=\frac{J'_{y_2}}{n_\mathrm{GJ}}, \end{aligned}$$

and thus

176 $$\begin{aligned} G(x_0,y_k)=\frac{1}{n_\mathrm{GJ}}G'(x_0,y_2). \end{aligned}$$
